# Testing evolutionary explanations for the lifespan benefit of dietary restriction in fruit flies (*Drosophila melanogaster*)

**DOI:** 10.1111/evo.14146

**Published:** 2021-01-12

**Authors:** Eevi Savola, Clara Montgomery, Fergal M. Waldron, Katy M. Monteith, Pedro Vale, Craig Walling

**Affiliations:** ^1^ Institute of Evolutionary Biology, School of Biological Sciences The University of Edinburgh Ashworth Laboratories Edinburgh EH9 3FL UK

**Keywords:** Ageing, bacteria, diet, dietary restriction, *Drosophila melanogaster*, infection

## Abstract

Dietary restriction (DR), limiting calories or specific nutrients without malnutrition, extends lifespan across diverse taxa. Traditionally, this lifespan extension has been explained as a result of diet‐mediated changes in the trade‐off between lifespan and reproduction, with survival favored when resources are scarce. However, a recently proposed alternative suggests that the selective benefit of the response to DR is the maintenance of reproduction. This hypothesis predicts that lifespan extension is a side effect of benign laboratory conditions, and DR individuals would be frailer and unable to deal with additional stressors, and thus lifespan extension should disappear under more stressful conditions. We tested this by rearing outbred female fruit flies (*Drosophila melanogaster*) on 10 different protein:carbohydrate diets. Flies were either infected with a bacterial pathogen (*Pseudomonas entomophila*), injured with a sterile pinprick, or unstressed. We monitored lifespan, fecundity, and measures of aging. DR extended lifespan and reduced reproduction irrespective of injury and infection. Infected flies on lower protein diets had particularly poor survival. Exposure to infection and injury did not substantially alter the relationship between diet and aging patterns. These results do not provide support for lifespan extension under DR being a side effect of benign laboratory conditions.

Nutrition has long been of interest in the field of aging research, particularly due to its potential applications to an aging human population (reviewed in Redman and Ravussin 2011; Speakman and Mitchell 2011; Bertozzi et al. 2016). Dietary restriction (DR), the limitation of a particular nutrient or the overall caloric intake without malnutrition, has been shown to extend lifespan and delay aging across a range of organisms (reviewed in Mair and Dillin 2008). Its prevalence and taxonomic diversity suggest the response is evolutionarily conserved and acts via conserved mechanisms (reviewed by Fontana et al. 2010). As such, a large body of research has focused on using the DR paradigm to try to understand the mechanisms underlying variation in aging and lifespan (e.g., Gems and Partridge 2012; Fontana and Partridge 2015; Gibbs and Smith 2016). However, the evolutionary basis of the response has been much less well investigated (Raubenheimer et al. 2016; Zajitschek et al. 2016; Moatt et al. 2020; Regan et al. 2020; Travers et al. 2020). This is surprising given that knowledge of the evolutionary basis of the DR response is important to understanding under what conditions it may be applicable in human health. Here we test the two main evolutionary explanations for lifespan extension under DR, which make contrasting predictions about how this response should vary across environments.

The predominant evolutionary explanation, termed the resource reallocation hypothesis (RRH) (Adler and Bonduriansky 2014; Regan et al. 2020), explains the observed DR response as an adaptive shift in relative investment of resources into survival versus reproduction (Kirkwood 1977; Shanley and Kirkwood 2000; Adler and Bonduriansky 2014). A food shortage signals a sub‐optimal environment, where the number and survival probability of any offspring produced is likely to be low (Holliday 1989; Shanley and Kirkwood 2000). Under such conditions, an individual could maximize fitness by temporarily delaying reproduction and instead investing resources into survival and somatic maintenance. Once food availability returns, the individual could then maximize fitness by investing resources back into reproduction. By maintaining individuals chronically on low food, aging rates decrease and the individual lives longer (Holliday 1989; Shanley and Kirkwood 2000). The RRH requires a trade‐off between investing resources into reproduction versus somatic maintenance (Holliday 1989) and that the response evolved in an environment that fluctuates between low and high food availability (Adler and Bonduriansky 2014).

In contrast to the predictions of the RRH, some studies suggest that survival and reproduction can be uncoupled under DR (reviewed in Flatt 2011). In addition, wild systems have much higher levels of extrinsic mortality than laboratory conditions (for example, from predators or disease), potentially making an individual less likely to live long enough to benefit from delayed reproduction (Adler and Bonduriansky 2014). These observations have been used to suggest that improved survival may not be the selective benefit of the DR response (Adler and Bonduriansky 2014). Instead, another hypothesis proposes that the selective benefit of the DR response is through its effect on immediate reproduction (Adler and Bonduriansky 2014), termed the nutrient recycling hypothesis (NRH) (Regan et al. 2020). This hypothesis is based on the general finding that DR results in the inhibition of nutrient‐sensing pathways, e.g. TOR and IIS pathways (Adler and Bonduriansky 2014). Inhibition of these pathways disinhibits (upregulates) nutrient recycling mechanisms such as apoptosis (James et al. 1998) and autophagy (Hansen et al. 2008; Kenyon 2010; Fontana et al. 2010, both reviewed in Longo and Fontana 2010). The NRH suggests that apoptosis and autophagy allow the organism to use stored nutrients from cells whilst limiting the number of cells (Adler and Bonduriansky 2014). The individual can use available resources more efficiently, with a possible lower resource requirement for reproduction (Adler and Bonduriansky 2014).

The NRH posits that lifespan extension under DR is an artifact of laboratory conditions. Upregulation of apoptosis and autophagy may promote survival and limit rates of aging due to protecting against common laboratory causes of death, such as cancer or other old age pathologies (Zhang and Herman 2002; Spindler 2005; Salomon and Jackson 2008; Longo and Fontana 2010; Adler and Bonduriansky 2014). However, the limit on cell numbers and cellular growth rate may also limit the ability of individuals under DR to respond to additional stresses (Adler and Bonduriansky 2014), with the prediction that DR would not extend lifespan in the wild (Adler and Bonduriansky 2014). Thus, in contrast to the RRH, there is a clear prediction from the NRH that the addition of stressors, particularly injury and infection, should result in the removal or even reversal of the lifespan benefit of DR (Adler and Bonduriansky 2014).

The effect of DR has been subject to relatively few studies in the context of injury and infection stress. In terms of injury stress, decreased calorie intake slows down wound repair in both rodents and reptiles (Reiser et al. 1995; Reed et al. 1996; French et al. 2007; Hunt et al. 2012). However, studies manipulating both overall calories and macronutrient content suggest that the main driver of the DR response, particularly in insects, is macronutrient ratio, with low protein and high carbohydrate diets leading to longer lifespans (e.g., Le Couteur et al. 2016; Lee et al. 2008; Simpson and Raubenheimer 2009; Nakagawa et al. 2012). In terms of infection stress, evidence for protein to carbohydrate (P:C) ratio effects on proxies of survival after infection are mixed. In infected caterpillars, higher protein increases performance, measured as the product of weight gain and survival to pupation (Lee et al. 2006; Povey et al. 2009, Povey et al. 2014), and lengthens the time to death for caterpillars dying post‐infection prior to pupation (Cotter et al. 2019; Wilson et al. 2020). In adult fruit flies (*Drosophila melanogaster*), higher protein increased survival 24 h post‐infection with bacterial infection (Kutzer et al. 2018) and higher protein as extra yeast on top of food increased the number of days alive post‐infection with a fungal pathogen (Le Rohellec and Le Bourg 2009). In contrast, higher protein decreased survival measured up to 160 h post‐infection (Lee et al. 2017), 16 days post‐infection in *D. melanogaster* (Ponton et al. 2020), and decreased survival 9 days post‐infection in Queensland fruit flies (*Bactrocera tryoni*; Dinh et al. 2019). However, to date, none of these experiments have directly measured the key trait of lifetime survival. Additionally, studies often only use a small number of diets (Le Rohellec and Le Bourg 2009; Lee et al. 2017; Kutzer et al. 2018; Dinh et al. 2019; Ponton et al. 2020), or manipulate both P:C and calories at the same time (Le Rohellec and Le Bourg 2009; Lee et al. 2017; Kutzer et al. 2018), making it hard to disentangle that aspect of the diet is affecting survival with injury or infection. Furthermore, no experiments have directly compared the effect of multiple diets on lifetime survival and reproduction in control, injured, and infected individuals and thus tested the alternative predictions of the current evolutionary explanations of the DR response.

Here, we address this gap in our knowledge by testing the contrasting predictions of the current evolutionary explanations of the DR response by including additional stressors of injury and infection to dietary restricted *D. melanogaster*. We achieved DR by altering the P:C ratio of food (e.g. Jensen et al. 2015; Lee et al. 2008) and thus throughout use the term protein restriction, although we acknowledge this also means the associated increase in carbohydrate, and changes in lipids and micronutrients. We measured lifespan, reproduction, and aging measures, specifically the maintenance of gut integrity and climbing ability. These measures of aging are often used to track treatment‐specific declines in function (e.g., Grotewiel et al. 2005; Martins et al. 2018) and allow us to measure whether aging is delayed with DR under all stress treatments. We predict that if the RRH explains DR responses, all treatments would see the usual pattern of DR, where decreasing protein increases survival up to a point and then survival declines again due to malnutrition (see review Mair and Dillin 2008). Regardless of the stress treatment, reproduction would increase with increasing protein, and aging would be delayed with lower protein. If the NRH explains DR responses, we would expect to see that with injury and infection, the lifespan increase expected under DR would disappear, and injured and infected flies would not have the usual hump shape response of lifespan to decreasing protein in the diet. In addition, infected or injured individuals would not show delayed aging with DR. Only the control group with no stress treatment would show the usual DR responses.

## Methods

### FLY STOCKS AND MAINTENANCE CONDITIONS

We used an outbred population of *D. melanogaster*, created by crossing 113 *Drosophila melanogaster* Genetic Reference Panel (DGRP; Mackay et al. 2012) lines in 100 pairwise crosses (consisting of two age‐matched virgin females and two age‐matched males from different DGRP lines; see supporting information) in vials containing modified Lewis food (Lewis 1960, see Table S1, 14% protein diet). The first generation of the outcross was made by placing all offspring from these initial pairwise crosses in a population cage and allowing them to interbreed and lay eggs on fruit juice agar plates. These eggs were collected by pouring PBS solution on the plates and collecting the egg solution in a falcon tube, which was then deposited into bottles containing Lewis food, following the method of Clancy and Kennington (2001) for maintaining *Drosophila* populations at constant densities. To generate the next generation, each month the emerged adult flies from these bottles were pooled into a population cage to lay eggs following the same method of Clancy and Kennington (2001; more information in supporting information). In this way, the outcrossed DGRP population was housed in plastic bottles and outbred for 19 non‐overlapping generations of complete outcrossing in 12 h light:dark cycles, at 25°C (±1°C) and constant humidity. Many of the original DGRP lines carry the bacterial endosymbiont *Wolbachia* (Mackay et al. 2012). The DGRP panel in the laboratory was cleared of *Wolbachia* over 7 years prior to the creation of the outcrossed population.

From the 20th overlapping generation of this outcrossed population, 4 μl of egg solution was placed into 20 plastic vials with modified Lewis food. After one generation, the adults were split into 50 vials, and to 60 vials from the second generation onward. To create each generation, adults were transferred to new vials and allowed to lay eggs for two days before removal. Flies used for the experiment were offspring of the fifth generation from this protocol. The DGRP outcrossed population tested negative for common *Drosophila* laboratory viruses using primers described in Webster et al. (2015) with RT‐PCR (unpublished data).

### EXPERIMENTAL METHODS

Adults of the fifth generation were density controlled (10 females/vial) to minimize subsequent variation in larval densities across vials, which can affect adult life‐history traits (Graves and Mueller 1993). Mated females were allowed to lay eggs for two days before removal. Vials were checked daily for adult eclosion. Flies were then maintained in vials for five days after adult eclosion began to allow mating to occur after which mated female flies from over 30 of these vials were transferred into the experiment following handling under CO_2_ anesthetization. At this point, individual flies were singly housed on one of the ten diet treatments for the first experimental day (see below). On experimental day 2, flies from each diet treatment were assigned to one of three stress treatments: control, injury, or infection (see below). There were 20 replicate flies per diet and stress treatment combination (20 individuals × 3 treatments × 10 diets = 600 flies in total). Females from one of the 30 vials were included across diet and stress treatments to account for some of the potential variation from the larval or adult environment.

#### Diet treatments

For the adult lifespan of each fly, flies were maintained on one of ten diets varying in protein to carbohydrate (P:C) ratio. These diets were made by altering the mass of yeast or sugar added to the modified Lewis food recipe (Lewis 1960; Table S1). Protein percentages and P:C values incorporate protein and carbohydrate values from maize. Yeast contains various micronutrients and carbohydrates outside of protein (Simpson and Raubenheimer 2009; Lee 2015), however here yeast is considered only as a protein source due to lack of direct quantification of dietary protein and carbohydrate in the yeast used. The 10 diets were a span of P:C values (from 1:26 to 2.5:1 P:C), where protein restriction has previously been shown to extend lifespan (Lee 2015).

#### Stress treatments

On experimental day 2, flies were exposed to one of three stress treatments: control, injury or infection. The control treatment involved handling flies under CO_2_ anesthetization and then transferring these to a new vial containing the relevant diet. The injury treatment involved the same protocol, however, an enameled pin was dipped in sterile LB broth and used to pierce the pleural suture under the left wing. For the infection treatment, the pin was dipped in a *P. entomophila* bacterial broth from an overnight culture in LB at 30 °C (following Dieppois et al. 2015; Troha and Buchon 2019, see also Chakrabarti et al. 2012; Vodovar et al. 2005 for more information on the pathogen). To avoid lethal or negligible doses, an OD of 0.005 of *P. entomophila* culture was used, as determined in a previous pilot study (unpublished data).

### SURVIVAL AND FECUNDITY MEASURES

Individuals were followed for life with survival scored daily. For the first two weeks of the experiment, individuals were tipped into fresh vials daily, and afterward every second day, with eggs (hatched and unhatched) counted when tipped. Any additional eggs in the vial were counted if a fly died on a day without a scheduled egg count. Diets and stress treatments were randomized across trays and trays were moved around the incubator daily to minimize microclimate effects.

### MEASURES OF PHYSIOLOGICAL AGING

#### Gut deterioration (smurf) assay

In *D. melanogaster*, and other species (Martins et al. 2018), physiological aging is associated with increased gut permeability, which can be assessed by feeding flies food with a blue dye and observing a change in body color if the dye leaks from the gut (Rera et al. 2011). All diets included a blue food dye following Rera et al. (2011) at a lower concentration (Table S1), to allow individuals to be scored for the “smurf” phenotype with age (Rera et al. 2011). Flies were scored as smurfs if the full body was blue, rather than just a small amount in the abdomen (Rera et al. 2011).

#### Negative geotaxis (NG) assay

As flies age, their escape response declines and this deterioration can be measured with a negative geotaxis (NG) assay (e.g. Arking and Wells 1990; Gargano et al. 2005; Linderman et al. 2012). NG was measured once every two weeks from week three, with a method modified from Arking and Wells (1990, see supporting information). Briefly, flies were individually tipped into clean vials, knocked down to the bottom, and then scored for whether they climbed to 4 cm on the vial within 60 seconds (1 for passing line, 0 for not passing the line).

### STATISTICAL METHODS

The data were analyzed using R software, version 3.5.2 (R Core Team 2014) and graphs were drawn using ggplot2 (Wickham 2016). Diet was analyzed as a continuous covariate, representing the percentage of protein (Table S1), and its quadratic effect to allow for non‐linear effects, whereas stress treatment was analyzed as a categorical fixed effect. These and their interactions were included in all models. When reporting the results of the full models, the reported main effect represents the posterior mean and associated credible intervals for the baseline of control unstressed flies. For interactions, posterior means and associated credible intervals are the differences in slope for the specific effect in comparison to the control unstressed baseline (main effects in the model). To avoid scaling errors, all variables were standardized to a mean of zero with a standard deviation of one. This was done separately for each test due to different sample sizes. We used the R package MCMCglmm (Hadfield 2010) for all models using a Poisson error distribution, unless otherwise stated. Further details are included in supporting information.

We used the R Survminer package (Kassambara and Kosinski 2018) to graph Kaplan‐Mayer curves for each stress treatment with diet as a factor. Our survival data violated the Cox proportional hazards assumptions, so we used an event history model where survival was analyzed as a binomial trait, with each day scoring a fly as 0 for alive or 1 for dead, following Moatt et al. (2019). We included random effects of individual identity to account for repeated measures and experimental day to account for variation in survival across days. To confirm these results, we also analyzed lifespan (see supporting information for details). We analyzed lifetime reproduction and additionally, to remove the effect of lifespan, we included mean centered lifespan in a separate model. For easier comparisons to other studies, early egg production was analyzed (days 2–7, as first day counts were similar across diets (Figure S1)). To investigate reproductive senescence, daily egg counts were analyzed with age (in days) and its squared term as fixed effects and with mean‐centered lifespan as a fixed effect, to control for selective disappearance (Van de Pol and Verhulst 2006), and a random effect of individual identity was included. The binomial variable for appearance of a blue body (1 for smurf, 0 for none) was analysed with a categorical model. Negative geotaxis was analysed as a binomial variable (1 for passing test, 0 for not) with a categorical model. Data and associated script are available on the Dryad repository (Savola et al. 2020).

## Results

### SURVIVAL AND LIFESPAN

**Figure 1:**
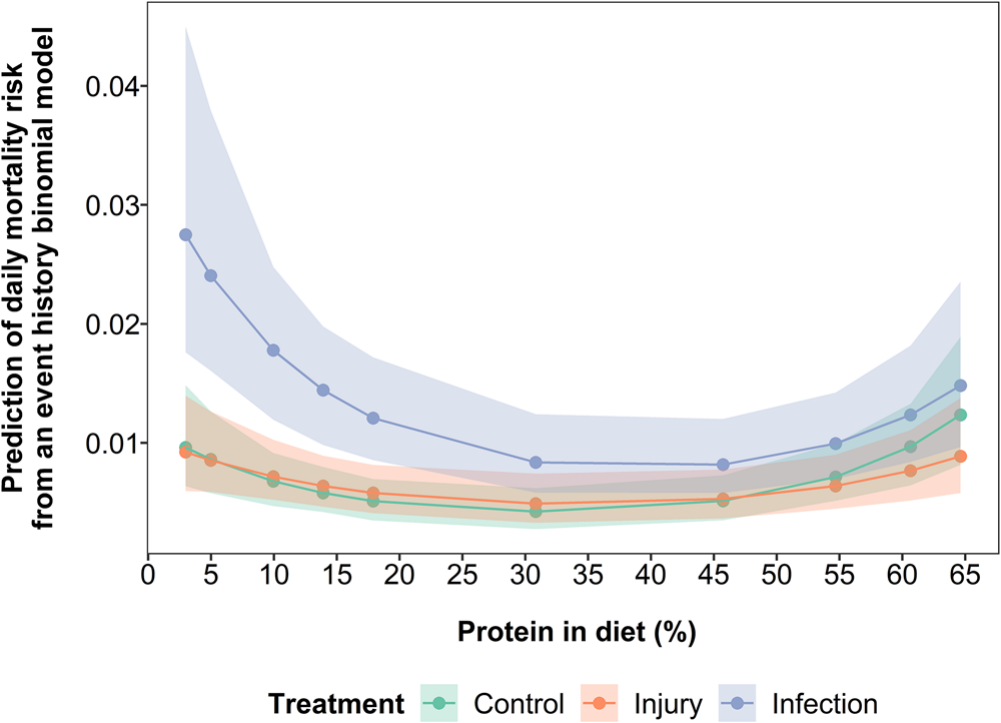
Model predictions from an event history binomial model for the effect of protein restriction on mortality risk per day of flies infected with a bacterial pathogen (blue data points and lines), injured by a pinprick (orange data points and lines) or with no treatment (green data points and lines). In the binomial model, for each day each fly was coded as 0 for alive and 1 for dead. Protein and protein^2^ are mean centered to standard deviation of 1. Shaded areas are 95% credible intervals.

Analysing the survival data with an event history binomial model, the improvement in survival with reduced protein from very high protein levels (i.e. the classical DR response in *D. melanogaster*) did not differ across treatments, and survival was maximised at relatively similar intermediate protein levels across treatments (Figure 1 and S2; Table S2; Protein^2^ = 0.48 (95% credible interval (CI) = 0.26 to 0.71), *p* ≤ 0.001; Injury:Protein^2^ = −0.16 (95% CI = −0.51 to 0.18), *p* = 0.36; Infection:Protein^2^ = −0.01 (95% CI = −0.33 to 0.30), *p* = 0.99). There was a significant interaction between protein and stress treatment, with survival increasing more rapidly from low to intermediate protein levels for the infected treatment than for any other treatment (Fig. 1 and S2; Table S2; Protein = 0.02 (95% CI = −0.13 to 1.17), *p* = 0.82; Infection:Protein = −0.31 (95% CI = −0.57 to −0.10), p = 0.004). This difference may be due to the low survival of infected individuals on low protein diets (Fig. S2).

Stress treatment had a significant effect on survival, with individuals exposed to the infection having a greater risk of death compared to control individuals for the duration of the experiment (Table S2; Infection = 0.66 [95% CI = 0.28 to 1.10] *p* = 0.002). There was no significant difference between injury and control treatments (Table S2; Injury = 0.14 (95% CI = −0.32 to 0.57), *p* = 0.54). Analyzing lifespan (in days) showed very similar patterns to the binomial survival analysis (Figs. S3 and S4; Table S3). Although our survival data violated the Cox proportional hazards model assumptions (see supporting information), the results from a Cox proportional hazards model were similar to those from the event history and lifespan models (Fig. S5; Table S4).

### REPRODUCTION

**Figure 2:**
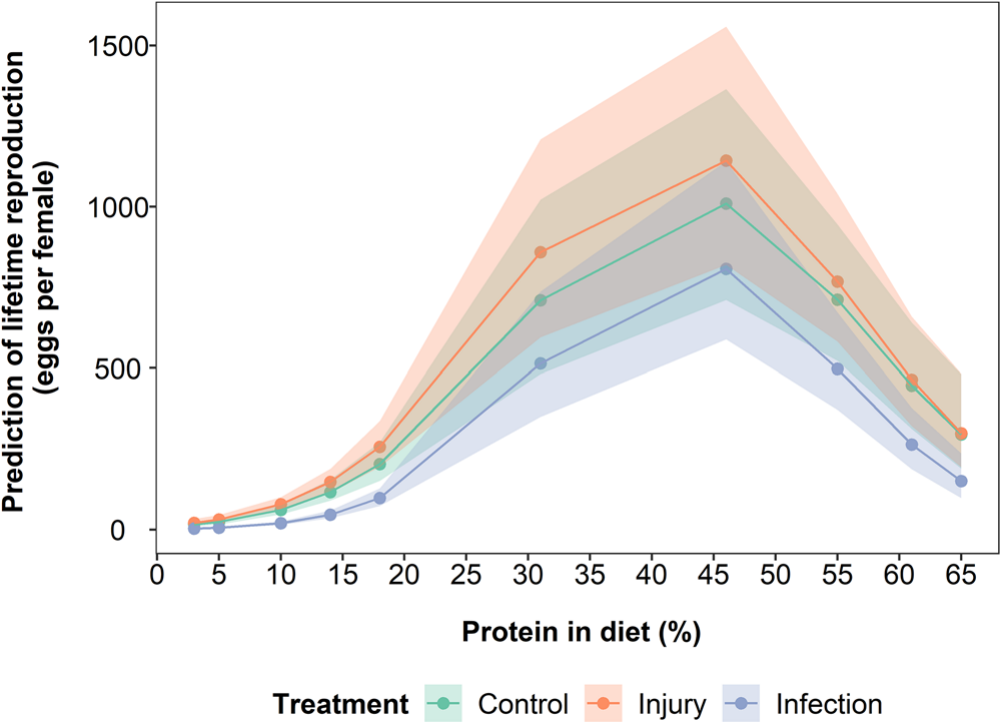
Model predictions of the effect of protein restriction on the lifetime egg production of flies infected with a bacterial pathogen (blue data points and lines), injured by a pinprick (orange data points and lines) or with no treatment (green data points and lines). Shaded areas are 95% credible intervals. Protein and protein^2^ are mean centered to standard deviation of 1.

Stress treatment had no significant effect on the lifetime number of eggs produced at mean levels of dietary protein (Table S5; Injury = 0.19 (95% CI = −0.34 to 0.72), *p* = 0.49; Infection = −0.33 (95% CI = −0.90 to 0.16), *p* = 0.26), but there was a significant interaction between stress treatment and both protein and its squared term (Table S5; Infection:Protein = 0.47 (95% CI = 0.16 to 0.77), *p* = 0.01; Infection:Protein^2^ = −0.47 (95% CI = −0.93 to −0.04), *p* = 0.04). For the baseline of control unstressed flies, lifetime egg production was highest at high but not the highest protein levels, with flies on low protein diets in particular producing very few eggs (Fig. 2; Fig. S6; Table S5; Protein = 1.45 (95% CI = 1.23 to 1.64), *p* ≤ 0.001; Protein^2^ = −1.36 (95% CI = −1.68 to −1.02), *p* ≤ 0.001). Therefore, the protein and stress treatment interactions suggest that infected individuals had a higher linear increase in lifetime eggs with increasing protein, but this relationship was also more curved, than in either the control or injury group. Despite these significant interactions, the broad pattern of change in egg counts with changing protein level is similar across stress treatments (Fig. 2).

To control for variation in lifetime egg production due to differences in lifespan, early‐life egg production was also analyzed. For eggs produced in the first week, excluding the first day, the patterns were similar to those of lifetime egg production (Figs. S6, S7, and S8; Tables S5 and S6). However, there was no interaction between stress treatment and protein on early‐life egg production (Fig. S8; Table S6; Infection:Protein = −0.24 (95% CI = −0.74 to 0.20), *p* = 0.32; Infection:Protein^2^ = −0.14 (95% CI = −0.85 to 0.57), *p* = 0.74). The decline in egg production at higher protein levels was reduced compared to lifetime egg production, such that early‐life egg production plateaus after reaching a maximum at intermediate protein levels, with a slight decline at very high protein levels (Fig. S8; Table S6; Protein^2^ = −0.86 (95% CI = −1.34 to −0.41), *p* ≤ 0.001). Similar patterns were seen in models of lifetime egg production with mean‐centered lifespan included in the model (Fig. S9; Table S7), suggesting that differences in lifetime reproduction between stress treatments are driven by the short lifespan of infected flies on low protein diets (Fig. 2; Figs. S8 and S9). As might be expected, flies with longer lifespans had more eggs over their life than shorter‐lived flies (Table S7, Lifespan = 0.93 (95% CI = 0.83 to 1.04), *p* ≤ 0.001).

### AGING

#### Daily egg production

**Figure 3:**
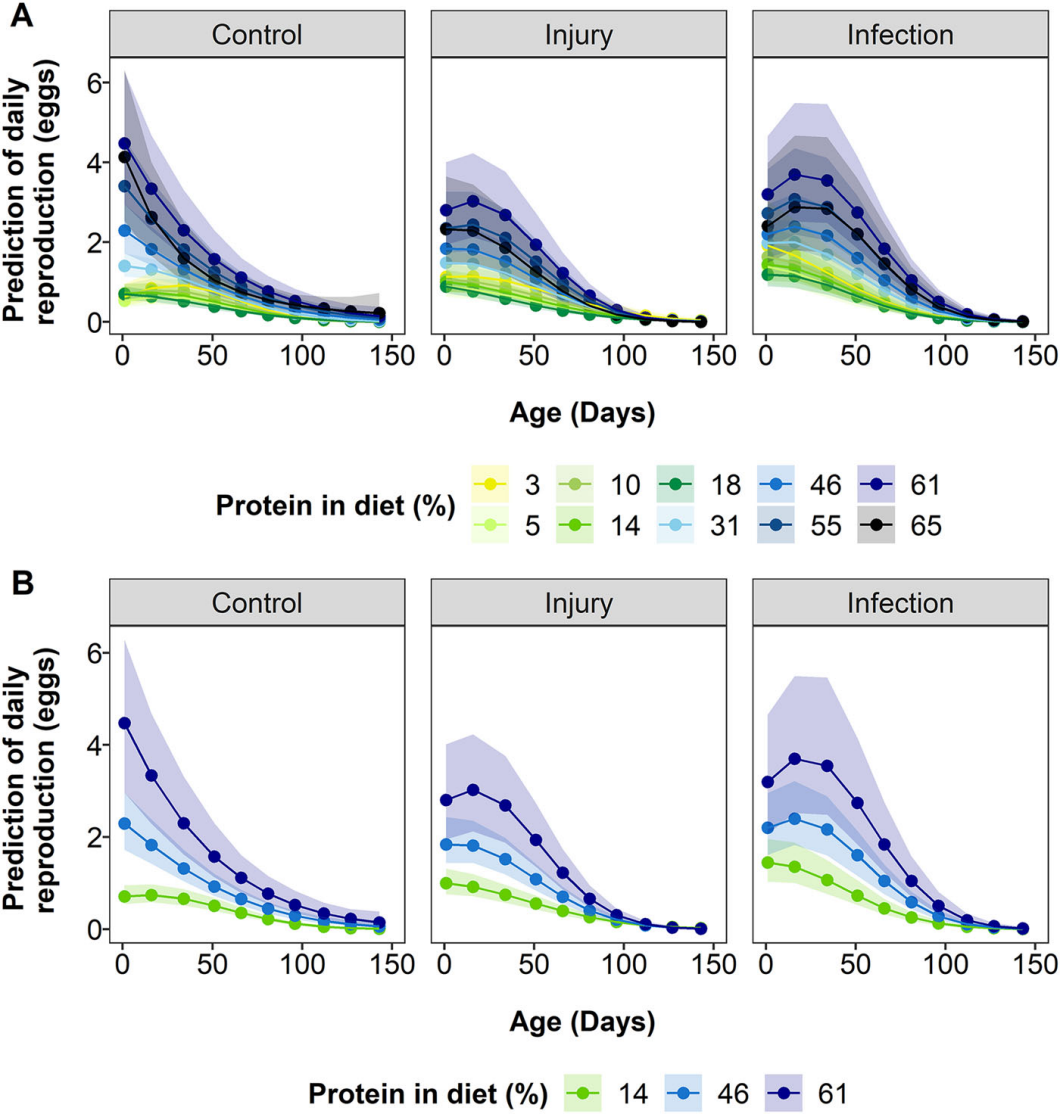
Model predictions of the effect of protein restriction and age on daily egg production of flies infected with a bacterial pathogen (“Infection”), injured by pinprick (“Injury”) or with no treatment (“Control”). Model predictions are shown for (A) all diets, or (B) for ease of interpretation, for a subset of protein restriction diets to illustrate the effects of protein restriction with low (green line), intermediate (light blue line) and high protein content (dark blue line). Shaded areas are 95% credible intervals. Protein, protein^2^ and lifespan are mean centered to standard deviation of 1.

There were numerous significant two‐ and three‐way interactions in the daily egg production model. Control unstressed individuals on average protein diets produced most eggs per day early in life, with significantly declining egg production with age (Fig. 3; Fig. S10; Table S8; Age = −0.32 (95% CI = −0.40 to −0.23), *p* ≤ 0.001), but this decline was nonlinear (Fig. 3; Fig. S10; Table S8; Age^2^ = −0.52 (95% CI = −0.59 to −0.44), *p* ≤ 0.001). With higher protein, control individuals were able to produce significantly more eggs per day (Fig. 3; Fig. S10; Table S8; Protein = 1.31 (95% CI = 1.12 to 1.52), *p* ≤ 0.001). However, at very low and high levels of protein, egg production reduced (Fig. 3; Fig. S10; Table S8; Protein^2^ = −1.5 (95% CI = −1.51 to −1.81), *p* ≤ 0.001). For these control unstressed individuals, the decline in reproduction with age is steepest and less curved at higher protein levels, but not at the highest protein levels (Fig. 3; Fig. S10; Table S8; Protein^2^:Age = −0.24 (95% CI = −0.32 to −0.16), *p* ≤ 0.001; Protein^2^:Age^2^ = 0.30 (95% CI = 0.22 to 0.38), *p* ≤ 0.001).

For infected individuals, the three‐way interactions suggest that the curved relationship between reproduction and age is greatest for individuals on intermediate to high (but not the highest) protein diets in comparison to the control flies (Fig. 3; Fig. S10; Table S8;, Infection:Protein^2^:Age = 0.22 (95% CI = 0.09 to 0.35), *p* = 0.005; Infection:Protein^2^:Age^2^ = −0.37 (95%' CI = −0.51 to −0.23) *p* ≤ 0.001). Injured individuals show similar patterns to infected individuals, but in terms of the curvature with age, this change compared to the control flies is generally less than for infected individuals (Fig. 3; Fig. S10; Table S8; Injury:Protein^2^:Age = −0.12 (95% CI = −0.23 to −0.23), *p* = 0.04; Injury:Protein^2^:Age^2^ = −0.13 (95% CI = −0.25 to −0.02), *p* = 0.03). There was a significant effect of lifespan on daily egg production, suggesting that longer‐lived individuals produced more eggs per day (Table S8; Lifespan = 0.21 (95% CI = 0.11 to 0.31), *p* ≤ 0.001).

#### Gut deterioration (smurf) assay

To assess gut integrity as a measure of aging, flies were fed blue food and were scored as a smurf if they turned blue due to the blue food leaking from the gut. Only 11.0% of flies (63/573, excluding censored flies) became smurfs throughout the experiment, so these results should be interpreted with some caution. There was a significant two‐way interaction between injury treatment and protein content, where the decline in the proportion of smurfs with increasing protein content was stronger in the injury treatment than in the control treatment (Fig. S12; Table S9; Protein = −0.75 (95% CI = −1.24 to −0.21), *p* = 0.004; Injury:Protein = −1.96 (95% CI = −4.07 to −0.11), *p* = 0.01). There was also a significant interaction between stress treatment and the quadratic effect of protein (Table S9; Injury:Protein^2^ = −2.09 (95% CI = −4.22 to −0.47), *p* = 0.14; Infection:Protein^2^ = −1.73 (95% CI = −3.13 to −0.37), *p* = 0.01). This suggests that in infected individuals, the proportion of smurfs peaked at intermediate protein levels and then declined at both high and low protein levels. As smurfs start appearing at a later‐life stage, low survival in the infected individuals on high and low protein diets may be driving this effect.

#### Negative geotaxis (NG) assay

By assessing escape response as a measure of aging, there were no differences between control, injured or infected flies in passing the test (Figs. S13 and S14; Table S10). Having controlled for lifespan, the proportion of flies passing the NG test declined more steeply with age on higher protein diets (Fig. S14, Table S10, Protein:Age = −0.78 (95% CI = −1.06 to −0.49), *p* ≤ 0.001). The likelihood of passing the test decreased with increasing protein (Table S10; Protein = −0.65 (95% CI = −1.01 to −0.32), *p* = < 0.001), but the rate of this decline slowed at the highest protein levels (Table S10; Protein^2^ = −0.70 (95% CI = −1.21 to −0.21), *p* = 0.01). Older flies were less likely to pass the test (Table S10; Age = −3.57 (95% CI = −4.04 to −3.07), *p* ≤ 0.001). There was an effect of selective disappearance, where longer‐lived individuals passed the test at a higher rate than individuals with shorter lifespans did (Table S10, Lifespan = 0.84 (95% CI = 0.64 to 1.02), *p* ≤ 0.001).

## Discussion

Our results provide a rare test of the predictions of two alternative evolutionary explanations for the commonly observed extension of lifespan in response to dietary restriction (DR). The nutrient recycling hypothesis (NRH) predicts that DR will not extend lifespan with the addition of injury and infection to the usually benign laboratory environment (Adler and Bonduriansky 2014). Alternatively, the resource reallocation hypothesis (RRH) does not make this prediction (Shanley and Kirkwood 2000). We applied two stressors and diets ranging in protein to carbohydrate (P:C) ratios to a population of outbred female *Drosophila melanogaster* to test these predictions. Our data showed that lifespan extension and delayed aging with DR remained even with the addition of injury and infection, therefore supporting the RRH. In particular, survival and lifespan were maximized at intermediate protein levels and declined at very high and low protein levels across all stress treatments, typical of the DR response through P:C ratios (Carey et al. 2008; Skorupa et al. 2008; Lee 2015) or through other methods of DR (e.g., Bishop and Guarente 2007; Clancy et al. 2002; Lee et al. 2006; Magwere et al. 2004; Pletcher et al. 2005, see also meta‐analysis Nakagawa et al. 2012). It should be noted that our results reflect broad changes in protein through changes in yeast:sugar, as in many other studies in *D. melanogaster* (e.g., Lee et al. 2008; Skorupa et al. 2008; Bruce et al. 2013). Therefore, these effects may be a direct result of changes in micronutrients or specific amino acids (e.g., Simpson et al. 2015; Piper et al. 2017; Zanco et al. 2020).

A small number of other studies have also considered predictions from the NRH using alternative approaches to the ones used here. One tested the prediction that reproduction should decline if autophagy is inhibited under DR, but found that this was not the case in *Caenorhabditis elegans* (Travers et al. 2020). An experimental evolution study in *D. melanogaster* males hypothesized that according to the NRH, individuals under DR should be more efficient at using the available resources, and thus under long‐term DR, experimental evolution lines should evolve to have higher reproductive performance and increased survival with DR (Zajitschek et al. 2016). Against their predictions, there was no change in survival, although the DR selection lines did have higher reproductive performance (Zajitschek et al. 2016). A recent study using wild and captive antler flies found that protein restriction lowered mortality rate even in non‐laboratory conditions (Mautz et al. 2019), contradicting the suggestion of the NRH that DR would have no benefit in the wild due to higher extrinsic mortality rate and stressors (Adler and Bonduriansky 2014). This pattern was only present in one of the two years included in the study, highlighting the need for further studies. In general, it appears that the predictions of the NRH are not being met in the studies conducted to date (Adler and Bonduriansky 2014).

Although the pattern of a tent‐shaped response of survival and lifespan to increasing levels of protein restriction seen here is typical of many other studies (Carey et al. 2008; Skorupa et al. 2008; Lee 2015; Jang and Lee 2018; Kim et al. 2020), it does contrast with recent studies suggesting lifespan is maximized on diets with very low P:C (Lee et al. 2008; Maklakov et al. 2008; Fanson et al. 2009, 2012; Harrison et al. 2014; Solon‐Biet et al. 2014; Jensen et al. 2015). These studies use a nutritional geometry approach where diets that vary in both calories and macronutrient ratio are used to separate the effects of these two variables. One reason our results may differ is the difference in the delivery of the diets, as most nutritional geometry studies in *D. melanogaster* have used liquid diets that allow fine scale measures of intake, but result in very low survival rates across all diets (Lee et al. 2008; Jensen et al. 2015). Studies using solid diets with *D. melanogaster* have found greater lifespans than in the liquid diet results, and have often found that lifespan was not maximised at the lowest protein diets (Skorupa et al. 2008; Bruce et al. 2013; Jang and Lee 2018; Kim et al. 2020, but see results for males in Kim et al. 2020). This suggests that diet delivery may have effects on survival, at least in *D. melanogaster* (see Maklakov et al. 2008; Fanson et al. 2009, Fanson et al. 2012; Harrison et al. 2014; Solon‐Biet et al. 2014 for other species and work involving solid diets). More work is needed to understand the causes of the differences in lifespan between studies.

An alternative consideration that may explain why we did not see the highest lifespans at the lowest protein diets might be due to the fact that the stock diet in our laboratory is a 14% protein diet, which is a relatively low protein diet. In an experimental evolution study in *D. melanogaster*, females from low protein experimental selection lines no longer had increased lifespan with protein restriction in comparison to females from control selection lines (Zajitschek et al. 2019). In our experiment, the outcrossed DGRP population had no increased lifespan with diets lower in protein than the 14% protein. This suggests that lifespan being the maximized at intermediate rather than the lowest protein diets (as seen in some nutritional geometry studies above) may be reflective of previous dietary maintenance conditions.

Although survival was maximized at intermediate protein levels across all stress treatments, the survival of infected individuals on very low protein diets was particularly poor. A positive relationship between dietary protein content and survival when exposed to infection is a common finding (Table 1). This suggests that in general dietary protein is important for infection responses (e.g. Lee et al. 2006; Le Rohellec and Le Bourg 2009; Cotter et al. 2019). However, there are some exceptions to the pattern (Table 1). Other than several methodological differences between studies (Table 1, Lee et al. 2017; Miller and Cotter 2018; Dinh et al. 2019; Sieksmeyer et al. 2019; Ponton et al. 2020; Roberts and Longdon 2020), these differences may be driven by the particular host‐pathogen pair, as diet alters various components of the host response and pathogen performance, and these relationships vary between systems (e.g., Lee et al. 2006; Povey et al. 2009, Povey et al. 2014; Miller and Cotter 2018; Cotter et al. 2019; Wilson et al. 2020). Further evidence for host‐pathogen‐specific effects of diet comes from a meta‐analysis of the effect of host nutrition on pathogen virulence, which found both positive or negative effects on virulence depending on the system (Pike et al. 2019). To understand the relationship between dietary protein and the response to infection, further work across multiple hosts and pathogens combining multiple measures of both host and pathogen are needed.

**Table 1:** Effects of protein manipulation on survival with temperature or infection stressors in insect studies. For dietary manipulation, “Yeast restriction” is used when only yeast was restricted, so this dietary manipulation consisted of reduced calories and protein. For diets with higher survival with stressor, upwards arrows (↑) indicate survival was higher on higher protein diets, downwards signs (↓) indicate survival was higher on lower protein diets, and an equal sign ( = ) indicates that diet had no effect on survival post‐infection

Stressor	Species	Dietary protein manipulation	Survival measure	Diets with higher survival with stressor	References
Increasing temperature	*Drosophila melanogaster*	P:C	Lifespan	↓	Kim et al. 2020
Infection	*Drosophila melanogaster*	Addition of yeast on top of food	Number of days alive	↑	Le Rohellec and Le Bourg 2009
		Yeast restriction	24 hours	↑ /=	Kutzer et al. 2018
		P:C or yeast restriction	Up to 160 hours	↓	Lee et al. 2017
		P:C	16 days	↓	Ponton et al. 2020
	27 species of Drosophilidae	P:C	20 days	=	Roberts and Longdon 2020
	*Bactrocera tryoni* Queensland fruit flies	P:C (liquid)	9 days	↓	Dinh et al. 2019
	*Blatta orientalis* cockroaches	P:C	6 days	=	Sieksmeyer et al. 2019
	*Nicrophorus vespilloides* burying beetles	Protein:fat	22 days	↓	Miller and Cotter 2018
	*Spodoptera littoralis* caterpillars	P:C	Larval performance	↑	Lee et al. 2006
		P:C	Increased time to death	↑	Cotter et al. 2019; Wilson et al. 2020
	*Spodoptera exempta* caterpillars	P:C	Larval performance	↑	Povey et al. 2009, 2014

Lifetime reproduction was maximized at intermediate protein levels, although at a slightly higher protein level than lifespan, a result which has been seen in other studies (Lee et al. 2008; Harrison et al. 2014; Jensen et al. 2015, but see Carey et al. 2008; Moatt et al. 2019). The decline in egg production with higher protein was not as steep in the early‐life model, or in the model accounting for lifespan. Regardless of stress treatment, we saw the same patterns of the highest egg counts on intermediate protein. Infection reduced egg production, as seen in many studies focusing on the reproduction‐immunity trade‐off (reviewed in Schwenke et al. 2016). If lifetime reproduction models included lifespan, or only early‐life reproduction was considered, there was no difference in reproduction between the stress treatments. This suggests that the pattern of lower lifetime reproduction in infected flies is most likely due to infected flies having shorter lifespans. Similar to our results, yeast restriction in *D. melanogaster* had a larger effect on early‐life egg production than infection (Kutzer and Armitage 2016; Kutzer et al. 2018). Contrary to our results, immune response activation can reduce reproduction when diet is limited (Stahlschmidt et al. 2013; Hudson et al. 2019), for example, oral infection with *Pseudomonas aeruginosa* increased early‐life egg production but only on higher protein diets (Hudson et al. 2019). Therefore, the methods of infection or the particular host‐pathogen system may have an effect on the response of host reproduction on different diets.

The patterns of reproductive aging involved complex interactions between diet and stress treatment. Broadly, there were similar aging patterns across treatments and diets, with an increase in egg production followed by a peak and then diminishing egg numbers, as seen in other experiments (Carey et al. 2008; Le Rohellec and Le Bourg 2009). These peaks were higher for the high protein diets (but not necessarily the highest), most likely due to the requirement of protein for egg production (Wheeler 1996; Mirth et al. 2019). Diets with low protein (e.g., 3% to 18% protein) had the slowest rate of decline in egg production with age. This could simply be a result of individuals on high protein diets having much higher egg production earlier in life and thus a greater potential decline than on low protein diets. Individuals on high protein diets declined rapidly in egg production early in life before the rate of decline reduced to that of individuals on lower protein diets later in life, suggesting there is an initially higher rate of aging on higher protein diets. Additionally, the control flies had a more linear decline in egg laying, suggesting that injury and infection might slightly delay egg production. Previous studies have also found aging in female reproduction was quicker on higher protein with various diet manipulations (Carey et al. 2008; Le Rohellec and Le Bourg 2009; Jensen et al. 2015; Moatt et al. 2019, but see Maklakov et al. 2009). Overall, these similarities across studies suggest diet interacts with reproductive aging in a broadly similar way across species.

Other than aging in reproduction, we also investigated aging in traits that are not implicated in the survival‐reproduction trade‐off, as delayed aging is a known DR response (e.g., Ingram et al. 1987; Mattson et al. 2001; Le Rohellec and Le Bourg 2009; Rera et al. 2012; Regan et al. 2016). Aging in negative geotaxis (NG) was delayed on lower protein diets, as has been found in another study limiting the addition of live yeast on food (Le Rohellec and Le Bourg 2009). We did not see the effects of stress treatment on NG, in contrast to a study where infection reduced the NG response in one of two tested *D. melanogaster* genetic backgrounds (Linderman et al. 2012), suggesting variation in the response depending on the genetic background of the host. Given the flies used in our study are genetically heterogeneous, the patterns we observe should be representative of the average genotype in this population.

We also measured the loss of gut integrity of flies with age using a smurf assay, which has been found to be more common in flies on unrestricted diets (Rera et al. 2012; Regan et al. 2016). Unexpectedly, we saw higher numbers of smurfs with lower protein in the control and injury treatments, whereas in the infected treatment, we saw higher numbers at intermediate protein levels. One explanation is that the lowest protein diets may represent malnourished conditions, leading to an increase in the number of smurfs. Nonetheless, we would still expect a reduction in smurf numbers at intermediate protein. In addition, for infected flies, the high mortality at high and low protein levels may result in flies dying before reaching the age where smurfs start appearing. As *P. entomophila* oral infection is known to damage the gut (Chakrabarti et al. 2012; Dieppois et al. 2015), further work is required to understand why some infected individuals did not develop into smurfs. The major problem with the interpretation of these results is the very low number of smurfs, meaning these patterns may not be robust. We analyzed the smurf trait as a binary variable, however, smurfs can be scored as a continuous trait as all individuals develop the trait (Martins et al. 2018). By measuring the phenotype with only clear smurfs counted, we may have missed some more subtle patterns. More work is required to understand how the relationship between protein restriction and the appearance of smurfs varies with exposure to injury and infection.

## Conclusion

The addition of injury and infection did not remove the lifespan benefit of protein restriction or the delay in reproductive aging. Our study therefore provides no evidence to support the nutrient recycling hypothesis of the lifespan response to dietary restriction. Even though there were minor differences between stress treatments in the relationship between protein content of the diet and survival, the major pattern of survival being maximized at intermediate protein levels was maintained across stress treatments. With infection, survival was particularly poor on the lowest protein diets, whilst in the other treatment groups this difference was not as dramatic. The explanation for this pattern requires further investigation. Our results and those of other studies suggest that the resource reallocation hypothesis remains the best‐supported evolutionary explanation for the lifespan benefit of dietary restriction.

## AUTHOR CONTRIBUTIONS

E.S., P.V., and C.W. designed the experiment. E.S. and C.M. did the study, with E.S. completing data collection for the full experiment. F.M.W. and K.M.M. created and maintained the fly population used in the study and wrote the supplementary methods part for the creation of the fly population. E.S. analyzed the data with help from C.W. E.S. wrote the paper with help from C.W., P.V., C.M, F.M.W., and K.M.M.

## CONFLICTS OF INTEREST

The authors declare no conflicts of interest.

## DATA ARCHIVING

Data and associated script are available on the Dryad repository: https://doi.org/10.5061/dryad.80gb5mkq2.

Associate Editor: K. McGuigan

Handling Editor: M.L. Zelditch

## Supporting information


**Table S1**: Ten diets and their corresponding P:C ratios with additional information of each added ingredient.
**Figure S1**: Average eggs per day produced in the first week for each protein restriction diet of flies infected with a bacterial pathogen (“Infection”), injured by a pinprick (“Injury”) or with no treatment (“Control”).
**Figure S2**: Effects of protein restriction on survival of flies infected with a bacterial pathogen (“Infection”), injured by a pinprick (“Injury”) or with no treatment (“Control”).
**Table S2**: Model summary of effects of protein restriction and stress treatments on mortality risk per day from an event history binomial model.
**Figure S3**: Effects of protein restriction on the lifespan of flies infected with a bacterial pathogen (blue bars and data points), injured by a pinprick (orange bars and data points) or with no treatment (green bars and data points).
**Figure S4**: Model predictions of the effects of protein restriction on lifespan of flies infected with a bacterial pathogen (blue data points and lines), injured by a pinprick (orange data points and lines) or with no treatment (green data points and lines).
**Table S3**: Model summary of effects of protein restriction and stress treatments on lifespan.
**Figure S5**: Model predictions for the effects of protein restriction on survival of flies infected with a bacterial pathogen (blue data points and lines), injured by a pinprick (orange data points and lines) or with no treatment (green data points and lines).
**Table S4**: Cox proportional hazard regression model summary of effects of protein restriction and stress treatments on survival (n = 600, number of deaths = 573, concordance = 0.662, R2 = 0.142, Wald test = 97.98).
**Figure S6**: Effect of protein restriction on the lifetime egg production of flies infected with a bacterial pathogen (blue lines and data points), injured by a pinprick (orange lines and data points) or with no treatment (green lines and data points).
**Table S5**: Model summary of effects of protein restriction and stress treatments on lifetime eggs produced.
**Figure S7**: Effect of protein restriction on the early‐life egg production of flies infected with a bacterial pathogen (blue lines and data points), injured by a pinprick (orange lines and data points) or with no treatment (green lines and data points).
**Figure S8**: Model predictions of the effect of protein restriction on the early‐life egg production of flies infected with a bacterial pathogen (blue data points and lines), injured by a pinprick (orange data points and lines) or with no treatment (green data points and lines).
**Table S6**: Model summary of effect of protein restriction and stress treatment on early‐life egg production (first week discounting the first day, see methods).
**Figure S9**: Model predictions of the effects of protein restriction on the lifetime number of eggs produced by flies infected with a bacterial pathogen (blue data points and lines), injured by a pinprick (orange data points and lines) or with no treatment (green data points and lines), when accounting for lifespan (mean centred).
**Table S7**: Model summary of effects of protein restriction and stress treatments on lifetime eggs produced.
**Figure S10**: The pattern of ageing in egg production for each protein restriction diet for flies infected with a bacterial pathogen (“Infection”), injured by a pinprick (“Injury”) or with no treatment (“Control”).
**Table S8**: Model summary of effects of protein restriction, age and stress treatment for daily egg production on flies.
**Figure S11**: Effects of protein restriction on proportion of smurfs (blue bars) or no smurfs (green bars) across life of flies infected with a bacterial pathogen (“Infection”, N = 23), injured by a pinprick (“Injury”, N = 25) or with no treatment (“Control”, N = 15).
**Figure S12**: Model predictions of the effect of protein restriction on the proportion of flies developing into a smurf of flies infected with a bacterial pathogen (blue data points and lines), injured by pinprick (orange data points and lines) or with no treatment (green data points and lines). Protein and protein^2^ are mean centered to standard deviation of 1.
**Table S9**: Model summary of effects of protein restriction and stress treatment on proportion of flies developing into a smurf.
**Figure S13**: Effects of protein restriction on the proportion of flies passing the negative geotaxis test under 60 seconds per week of flies infected with a bacterial pathogen (“Infection”), injured by a pinprick (“Injury”), or with no treatment (“Control”) (A).
**Figure S14**: Model predictions of the effect of protein restriction and age on proportion passing negative geotaxis test under 60 seconds per week with flies infected with a bacterial pathogen (“Infection”), injured by pinprick (“Injury”) or with no treatment (“Control”).
**Table S10**: Model summary of effects of protein restriction, age and stress treatment for passing negative geotaxis test under 60 seconds. Protein, protein2, age, age2 and lifespan are mean centered to standard deviation of 1.
