# Supplementary material for: Testing evolutionary explanations for the lifespan benefit of dietary restriction in fruit flies (Drosophila melanogaster)
Source: Evolution. 2021 Jan 12;75(2):450–63. doi: 10.1111/evo.14146 (PMC8609428; doi:10.1111/evo.14146)
Supplement: Supplementary file 1 — Table S1: Ten diets and their corresponding P:C ratios with additional information of each added ingredient. Figure S1: Average eggs per day produced in the first week for each protein restriction diet of flies infected with a bacterial pathogen (“Infection”), injured by a pinprick (“Injury”) or with no treatment (“Control”). Figure S2: Effects of protein restriction on survival of flies infected with a bacterial pathogen (“Infection”), injured by a pinprick (“Injury”) or with no treatment (“Control”). Table S2: Model summary of effects of protein restriction and stress treatments on mortality risk per day from an event history binomial model. Figure S3: Effects of protein restriction on the lifespan of flies infected with a bacterial pathogen (blue bars and data points), injured by a pinprick (orange bars and data points) or with no treatment (green bars and data points). Figure S4: Model predictions of the effects of protein restriction on lifespan of flies infected with a bacterial pathogen (blue data points and lines), injured by a pinprick (orange data points and lines) or with no treatment (green data points and lines). Table S3: Model summary of effects of protein restriction and stress treatments on lifespan. Figure S5: Model predictions for the effects of protein restriction on survival of flies infected with a bacterial pathogen (blue data points and lines), injured by a pinprick (orange data points and lines) or with no treatment (green data points and lines). Table S4: Cox proportional hazard regression model summary of effects of protein restriction and stress treatments on survival (n = 600, number of deaths = 573, concordance = 0.662, R2 = 0.142, Wald test = 97.98). Figure S6: Effect of protein restriction on the lifetime egg production of flies infected with a bacterial pathogen (blue lines and data points), injured by a pinprick (orange lines and data points) or with no treatment (green lines and data points). Table S5: Model summary of effects of p [file EVO-75-450-s001.pdf]

SUPPLEMENTARY METHODS:

OUTCROSSED DGRP POPULATION*:* A genetically diverse laboratory population resource for *Drosophila melanogaster* studies

The outcrossed DGRP population was founded on 15/10/14, and is derived from 113 inbred DGRP lines sampled from a wild population in Raleigh, NC, USA (Mackay et al. 2012).

The outcrossed DGRP population was founded with genetic contributions from the following 113 DGRP lines; RAL-28, RAL-31, RAL-48, RAL-49, RAL-57, RAL-59, RAL-69, RAL-75, RAL-83, RAL-91, RAL-93, RAL-101, RAL-129, RAL-138, RAL-149, RAL-153, RAL-158, RAL-189, RAL-195, RAL-208, RAL-217, RAL-228, RAL-237, RAL-239, RAL-280, RAL-287, RAL-288, RAL-301, RAL-303, RAL-304, RAL-306, RAL-309, RAL-310, RAL-317, RAL-321, RAL-324, RAL-348, RAL-350, RAL-352, RAL-354, RAL-358, RAL-360, RAL-361, RAL-365, RAL-366, RAL-373, RAL-375, RAL-377, RAL-379, RAL-380, RAL-381, RAL-382, RAL-386, RAL-390, RAL-392, RAL-395, RAL-397, RAL-399, RAL-405, RAL-406, RAL-409, RAL-426, RAL-427, RAL-437, RAL-439, RAL-443, RAL-486, RAL-491, RAL-492, RAL-502, RAL-508, RAL-509, RAL-517, RAL-528, RAL-530, RAL-535, RAL-555, RAL-559, RAL-563, RAL-566, RAL-575, RAL-584, RAL-589, RAL-627, RAL-630, RAL-634, RAL-703, RAL-712, RAL-716, RAL-732, RAL-765, RAL-774, RAL-776, RAL-786, RAL-796, RAL-805, RAL-808, RAL-818, RAL-820, RAL-821, RAL-822, RAL-832, RAL-852, RAL-853, RAL-855, RAL-859, RAL-879, RAL-882, RAL-884, RAL-897, RAL-907, RAL-908, RAL-913.

To maximise the genetic contribution of each of the founder lines to the final outcrossed population, initial pairwise crosses between randomly selected population founder lines were carried out. The offspring from these pairwise crosses were then pooled into a population cage for

the 1st generation of outcrossing. Whilst a minimum of 57 pairwise crosses would encompass inclusion of all 113 founder lines, 100 pairwise crosses were carried out as a precautionary measure against a number of crosses failing to produce offspring (an upper limit of 100 was dictated by feasibility). Pairwise crosses were set up using two virgin females crossed to two males. All virgin females and males were age-matched controlled (1-6 days old when crosses were set up). Pairwise crosses were set up in standard Lewis medium containing vials and placed at 25°C for 5 days after which adults were removed.

For pairwise crosses, DGRP outcrossed population founder lines were randomly selected to contribute females or males for the following 100 crosses (scheme is "2 virgin females from line" x "2 males from line"): RAL-390 x RAL-381, RAL-280 x RAL-301, RAL-913 x RAL-365, RAL-796 x RAL-712, RAL-589 x RAL-49, RAL-350 x RAL-382, RAL-853 x RAL-158, RAL-288 x RAL-855, RAL-49 x RAL-366, RAL-303 x RAL-908, RAL-101 x RAL-303, RAL-712 x RAL-426, RAL-321 x RAL-732, RAL-377 x RAL-101, RAL-380 x RAL-879, RAL-820 x RAL-324, RAL-882 x RAL-535, RAL-439 x RAL-634, RAL-83 x RAL-409, RAL-28 x RAL-75, RAL-409 x RAL-832, RAL-879 x RAL-237, RAL-237 x RAL-239, RAL-443 x RAL-776, RAL-908 x RAL-627, RAL-59 x RAL-584, RAL-365 x RAL-796, RAL-634 x RAL-405, RAL-392 x RAL-852, RAL-129 x RAL-350, RAL-317 x RAL-306, RAL-427 x RAL-528, RAL-373 x RAL-502, RAL-386 x RAL-28, RAL-304 x RAL-392, RAL-774 x RAL-555, RAL-306 x RAL-386, RAL-310 x RAL-309, RAL-832 x RAL-287, RAL-405 x RAL-280, RAL-57 x RAL-774, RAL-627 x RAL-228, RAL-397 x RAL-821, RAL-348 x RAL-492, RAL-437 x RAL-443, RAL-91 x RAL-31, RAL-352 x RAL-575, RAL-301 x RAL-390, RAL-48 x RAL-897, RAL-575 x RAL-808, RAL-426 x RAL-373, RAL-375 x RAL-195, RAL-31 x RAL-59, RAL-897 x RAL-310, RAL-239 x RAL-486, RAL-287 x RAL-805, RAL-584 x RAL-765, RAL-381 x RAL-149, RAL-93 x RAL-

703, RAL-379 x RAL-517, RAL-821 x RAL-630, RAL-189 x RAL-853, RAL-399 x RAL-360, RAL-907 x RAL-217, RAL-535 x RAL-786, RAL-195 x RAL-395, RAL-852 x RAL-913, RAL-502 x RAL-818, RAL-361 x RAL-375, RAL-138 x RAL-491, RAL-808 x RAL-93, RAL-517 x RAL-208, RAL-153 x RAL-189, RAL-149 x RAL-352, RAL-732 x RAL-509, RAL-818 x RAL-563, RAL-630 x RAL-57, RAL-395 x RAL-380, RAL-358 x RAL-822, RAL-765 x RAL-406, RAL-703 x RAL-859, RAL-406 x RAL-153, RAL-508 x RAL-379, RAL-716 x RAL-427, RAL-509 x RAL-358, RAL-555 x RAL-48, RAL-360 x RAL-321, RAL-786 x RAL-69, RAL-855 x RAL-354, RAL-559 x RAL-437, RAL-563 x RAL-361, RAL-158 x RAL-559, RAL-805 x RAL-884, RAL-208 x RAL-566, RAL-492 x RAL-397, RAL-382 x RAL-399, RAL-75 x RAL-508, RAL-884 x RAL-138, RAL-530 x RAL-83, RAL-69 x RAL-348. Offspring from pairwise crosses were collected 28 days after parents were removed and pooled into a large *Drosophila* population cage, for the 1st generation of outcrossing and subsequent embryo collection.

For this, and each subsequent generation of outcrossing, the outcrossed DGRP population is maintained employing a method used to maintain constant larval densities (223 ± 14.3 (95% CI)) in stock bottles (Clancy and Kennington 2001). Briefly, this method involves populating a large *Drosophila* cage with thousands of flies on the day 1, providing these with fruit juice (grape/apple) agar plates for embryo laying. After a 24 hr habituation period, agar plates are replaced (day 2). On the day 3, agar plates are recovered and embryos are collected from the surface. Using PBS and a brush, concentrated egg/PBS solutions are prepared, and these are squirted on the surface of Lewis media in bottles. This process is typically carried out every 20-25 days. The outcrossed DGRP populations is maintained at a density of 20-25 bottles (20 bottles maintains the population at >4000 individuals).

DIETS:

Table S1: Ten diets and their corresponding P:C ratios with additional information of each added ingredient. The standard modified Lewis food (Lewis 1960) and associated P:C ratio is in bold. One of the main differences to the original Lewis food recipe is the replacement of dextrose and sucrose with brown sugar in our diets (Lewis, 1960). The P:C ratios (rounded to the nearest whole number) incorporate the protein and carbohydrate contributed by maize. Although yeast is also composed of carbohydrates, lipids and micronutrients (Simpson and Raubenheimer 2009; Lee 2015), here yeast is only considered as a source of protein, as we did not quantify the actual protein and carbohydrates present in the yeast used in our laboratory. Yeast and sugar are roughly isocaloric, so P:C ratios can be altered without altering the energy content of the diet by replacing yeast with sugar (Mair et al. 2005). Two baseline ratios were made with no addition of yeast (2.5:1) or sugar (1:26). All the diets were dyed using a food dye (brilliant blue FCF E133).

P:C ratio	% Protein	Yeast (g)	Sugar (g)	Maize (g)			Agar (g)	Nipagin (ml)	Food dye (g)	dH_2O (l)
				Total	Of which carbohydrate	Of which protein				
1:26	3	0.0	675.0	415	290.5	37.8	41.2	90	3	6
1:16	5	21.3	653.7	415	290.5	37.8	41.2	90	3	6
1:8	10	73.7	601.3	415	290.5	37.8	41.2	90	3	6
**1:6**	**14**	**112.5**	**562.5**	**415**	**290.5**	**37.8**	**41.2**	**90**	**3**	**6**
1:4	18	162.9	512.1	415	290.5	37.8	41.2	90	3	6
1:2	31	296.7	378.3	415	290.5	37.8	41.2	90	3	6
1:1	46	463.9	211.1	415	290.5	37.8	41.2	90	3	6
1.5:1	55	564.2	110.8	415	290.5	37.8	41.2	90	3	6
2:1	61	631.1	43.9	415	290.5	37.8	41.2	90	3	6
2.5:1	65	675.0	0.0	415	290.5	37.8	41.2	90	3	6

NEGATIVE GEOTAXIS (NG) ASSAY:

This assay quantifies the climbing response of flies in terms of distance or speed, following Arking and Wells (1990). A rubber band was tied 4 cm from the bottom around an empty vial. After the fly was tipped into this vial and blocked with a cotton bud, the vial was tapped down three times on a corkboard. The timer was started on the last tap and stopped once the fly fully crossed the line. After the test, the fly was transferred to a new food vial. An upper limit of 60 seconds was set as some flies did not climb or cross the line. One vial was used per fly to avoid confounding effects of reusing vials (Nichols et al. 2012) or possible spread of infection. Due to time of day effects (Gargano et al. 2005), testing order was reversed each week. If the fly did not touch the bottom of the vial, or if the timer was stopped incorrectly, a second trial was completed. Due to the number of failed tests where the fly did not cross the line (43% of 5,117 tests), negative geotaxis scores were analysed as a binomial variable for passing (1) or failing (0) the test in 60 seconds.

STATISTICAL METHODS:

SURVIVAL:

We used the R Survminer package (Kassambara and Kosinski 2018) to graph Kaplan-Mayer curves individually for each stress treatment with diet as a factor. We first analysed the survival data with a Cox proportional hazards model using the R Survival package (Therneau 2015). The model included protein content, its squared term, stress treatments and their interactions as fixed effects. The assumptions of a Cox proportional hazards model were violated (Therneau, 2015, cox.zph function global term Chi squared = 95.26, p = <0.001). Predicted risk

ratios for each diet and stress treatment were calculated using the predict function for the Cox proportional hazards model.

As our survival data did not follow the assumptions of a Cox proportional hazards model, therefore we used an event history model where survival was analysed as a binomial trait, with each day a fly scored as a 0 for being alive and 1 for dead, following Moatt et al. (2019). We used the R package MCMCglmm (Hadfield 2010) to model survival as a binomial variable with a categorical model. The model contained the fixed effects of stress treatment, protein content and its squared term (to model non-linear effects) and their interaction. Censored flies were included in the analysis (27 individuals, so 4.5% of the total), scoring a 0 until the day of censoring. A random effect of individual identity was included to account for repeated measures on the same individual and a random effect of experimental day was added to account for variation in survival across days. Parameter expanded priors were placed on all random effects ($V = 1$, $nu = 1$, $alpha.mu = 0$, $alpha.V = 1000$). The residual variance was fixed to 1, as it is inestimable in a binomial model. The model was run for 5,200,000 iterations, with a burnin of 1,200,000 iterations and a thinning interval of 4,000 iterations to minimise autocorrelation. Autocorrelation was checked from plots of the posterior distribution of all estimates for this and all subsequent models.

We also analysed lifespan to confirm the results of the survival analysis. Lifespan, the number of days an individual survived, was analysed using a generalised linear model with MCMCglmm. Censored flies were removed from the analysis. A Poisson family error distribution was assumed and the model was run for 65,000 iterations with a thinning interval of 50 iterations and a burnin of 15,000 iterations to minimise autocorrelation. Protein content, its squared term, stress treatments and their interactions were included as fixed effects. An inverse Gamma prior was placed on the residual variance ($V = 1$ and $nu = 0.002$).

REPRODUCTION:

Lifetime reproduction was measured as the sum of all eggs counted per female over her life. The effect of stress treatment, protein content, its squared term and their interactions were analysed using a MCMCglmm model with a Poisson error distribution. The model was run for 130,000 iterations, with a burnin of 30,000 iterations and a thinning interval of 100 iterations to minimise autocorrelation. An inverse Gamma prior was placed on the residual variance ($V = 1$ and $nu = 0.002$). To remove the effect of lifespan on reproduction, the same model with the effect of mean centered lifespan for each fly was analysed separately, except with 650,000 iterations, a burnin of 150,000 iterations and a thinning interval of 500. As an additional analysis to remove the effect of lifespan on reproduction and to compare our data with other studies using measures of early reproduction, early egg production was analysed separately. Egg counts from experimental day 2 (day after stress treatment) to day 7 were considered, as the first day egg counts were very low and were very similar across diets (Figure S1). Only individuals which lived to day 7 were considered. A MCMCglmm model with a Poisson error distribution was run with 260,000 iterations, a burnin of 60,000 iterations and a thinning interval of 200 iterations. An inverse Gamma prior was placed on the residual variance ($V = 1$ and $nu = 0.002$). The effect of stress treatment, protein content and its squared term were included in the model.

REPRODUCTIVE AGEING:

To investigate reproductive senescence, daily egg counts were analysed using MCMCglmm with a Poisson error distribution. When egg counts changed from daily to every second day counting, all values that correspond to eggs produced over two days were divided by two and rounded down to the nearest integer. Fixed effects included stress treatment, protein content and age (in days) and their squared terms, and all interactions. Mean centred lifespan was

included as a fixed effect to control for selective disappearance (Van de Pol and Verhulst 2006) and individual ID was included as a random effect to control for repeated measures on the same individual. Models were run for 2,600,000 iterations, with a thinning interval of 1,500 and a burnin of 600,000. A parameter expanded prior was used for the random effect of individual ($V = 1$, $nu = 1$, $alpha.mu$ = 0, $alpha.V = 1000$) and an inverse Gamma prior placed on the residuals ($V = 1$ and $nu = 0.002$).

GUT DETERIORATION (SMURF) ASSAY:

A fly was scored as a smurf if it developed a non-disappearing blue body appearance (1 for smurf, 0 for no smurf) at any point during its life. This binomial variable was analysed with a categorical model using MCMCglmm. This model included the fixed effects of stress treatment, protein content, its squared term and their interactions. Models were run for 26,000,000 iterations, with a thinning interval of 20,000 and a burnin of 6,000,000. The residuals variance was fixed to 1 as explained above.

NEGATIVE GEOTAXIS (NG) ASSAY:

We analysed the data from the negative geotaxis experiments as a binomial variable (1 for climbing 4 cm in 60 seconds, 0 for failing to do this) using a categorical family in MCMCglmm. Stress treatment, protein content and age and their squared terms, their interactions and mean centred lifespan were included as fixed effects and individual identity as a random effect. The model was run for 3,900,000 iterations, with a thinning interval of 3,000 and a burnin of 900,000. A parameter expanded prior was used for individual identity ($V = 1$, $nu = 1$, $alpha.mu = 0$, $alpha.V = 1000$) and the residual variance was fixed to 1 as explained above.

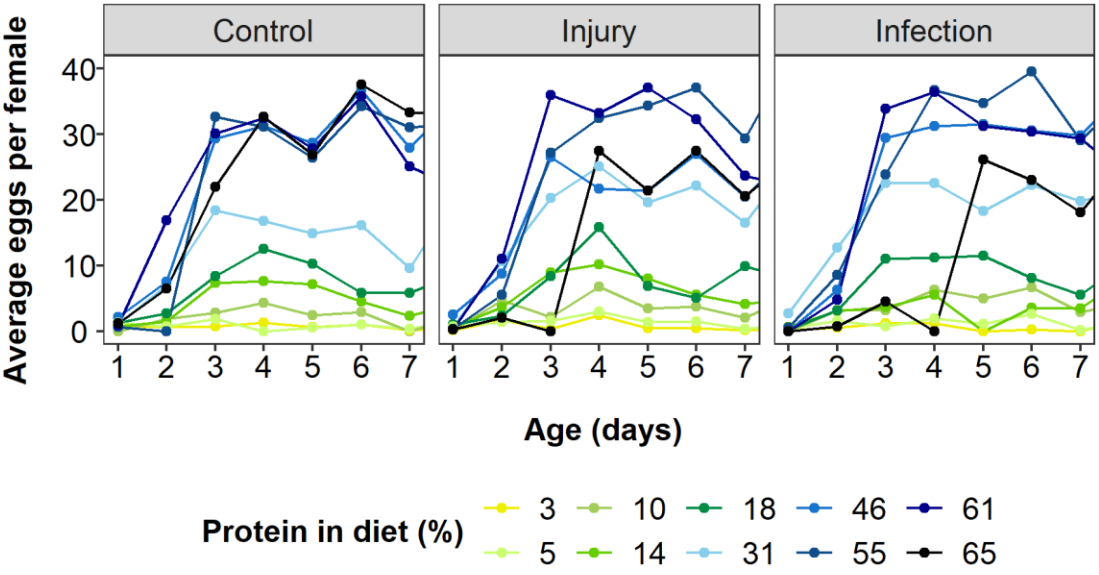

Figure S1: Average eggs per day produced in the first week for each protein restriction diet of flies infected with a bacterial pathogen ("Infection"), injured by a pinprick ("Injury") or with no treatment ("Control").

REFERENCES:

Arking, R., and R. A. Wells. 1990. Genetic alteration of normal aging processes is responsible for extended longevity in *Drosophila*. Dev. Genet. 11:141–148.

Clancy, D. J., and W. J. Kennington. 2001. A simple method to achieve consistent larval density in bottle cultures. Drosoph. Inf. Serv. 84:168–169.

Gargano, J. W., I. Martin, P. Bhandari, and M. S. Grotewiel. 2005. Rapid iterative negative geotaxis (RING): A new method for assessing age-related locomotor decline in *Drosophila*. Exp. Gerontol. 40:386–395.

Hadfield, J. D. 2010. MCMC Methods for Multi-Response Generalized Linear Mixed Models: The MCMCglmm R Package. J. Stat. Softw. 33:1–22.

Kassambara, A., and M. Kosinski. 2018. survminer: Drawing Survival Curves using "ggplot2."

Lee, K. P. 2015. Dietary protein:carbohydrate balance is a critical modulator of lifespan and reproduction in *Drosophila melanogaster*: A test using a chemically defined diet. J. Insect Physiol. 75:12–19.

Lewis, E. B. 1960. A new standard food medium. Drosoph. Inf. Serv. 34:117–118.

Mackay, T. F. C., S. Richards, E. A. Stone, A. Barbadilla, J. F. Ayroles, D. Zhu, S. Casillas, Y. Han, M. M. Magwire, J. M. Cridland, M. F. Richardson, R. R. H. Anholt, M. Barrón, C. Bess, K. P. Blankenburg, M. A. Carbone, D. Castellano, L. Chaboub, L. Duncan, Z. Harris, M. Javaid, J. C. Jayaseelan, S. N. Jhangiani, K. W. Jordan, F. Lara, F. Lawrence, S. L. Lee, P. Librado, R. S. Linheiro, R. F. Lyman, A. J. Mackey, M. Munidasa, D. M. Muzny, L. Nazareth, I. Newsham, L. Perales, L.-L. Pu, C. Qu, M. Ràmia, J. G. Reid, S. M. Rollmann,

J. Rozas, N. Saada, L. Turlapati, K. C. Worley, Y.-Q. Wu, A. Yamamoto, Y. Zhu, C. M. Bergman, K. R. Thornton, D. Mittelman, and R. A. Gibbs. 2012. The *Drosophila melanogaster* Genetic Reference Panel. Nature 482:173–178.

Mair, W., M. D. W. Piper, and L. Partridge. 2005. Calories do not explain extension of life span by dietary restriction in *Drosophila*. PLoS Biol. 3:1305–1311.

Moatt, J. P., M. A. Fyfe, E. Heap, L. J. M. Mitchell, F. Moon, and C. A. Walling. 2019. Reconciling nutritional geometry with classical dietary restriction: Effects of nutrient intake, not calories, on survival and reproduction. Aging Cell 18.

Nichols, C. D., J. Becnel, and U. B. Pandey. 2012. Methods to assay *Drosophila* behavior. J. Vis. Exp. e3795:1–5.

Simpson, S. J., and D. Raubenheimer. 2009. Macronutrient balance and lifespan. Aging (Albany. NY). 1:875–880.

Therneau, T. 2015. A Package for Survival Analysis in R.

Van de Pol, M., and S. Verhulst. 2006. Age-dependent traits: A new statistical model to separate within- and between-individual effects. Am. Nat. 167:766–773.

SUPPLEMENTARY RESULTS:

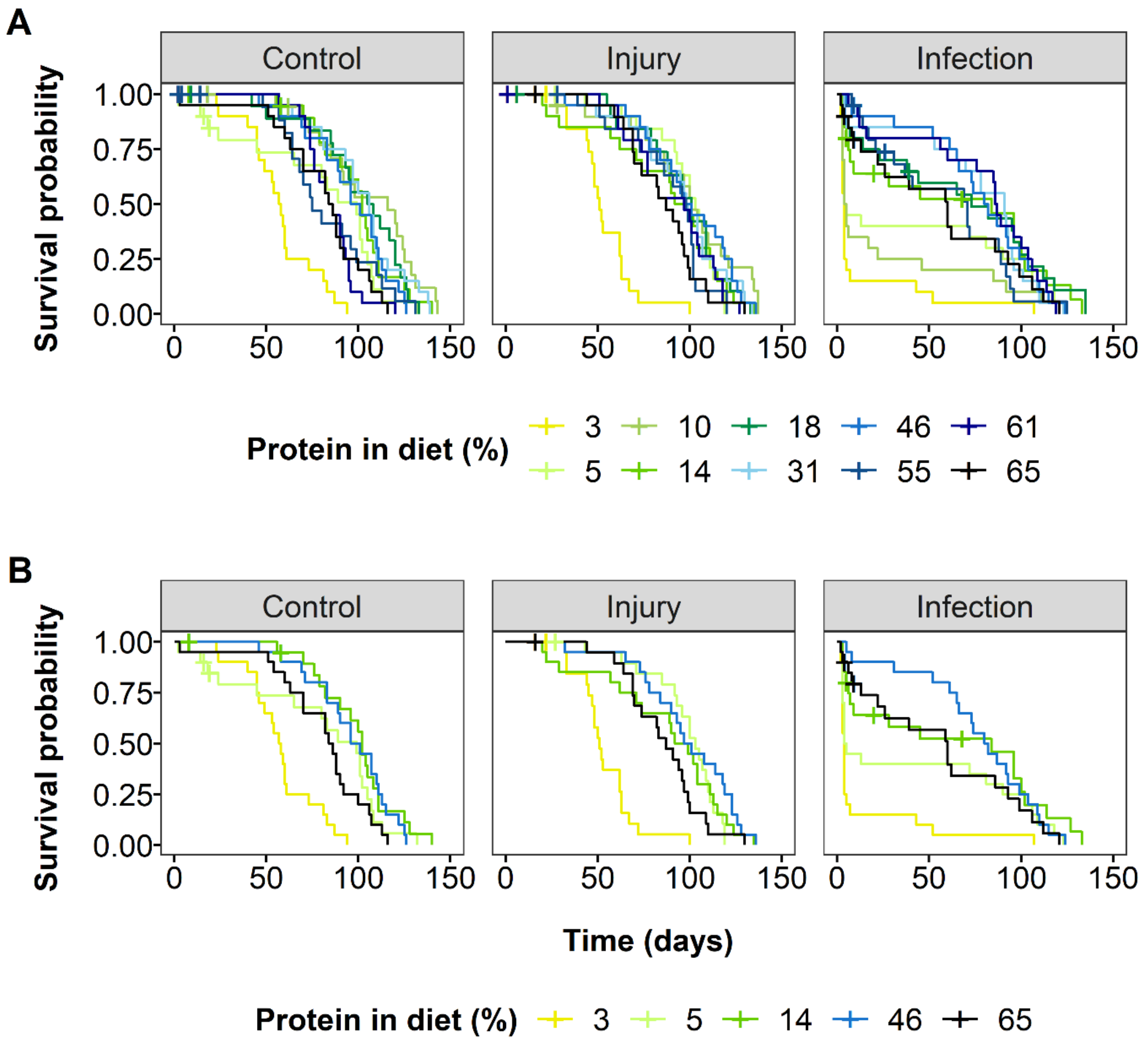

Figure 1: Effects of protein restriction on survival of flies infected with a bacterial pathogen ("Infection"), injured by a pinprick ("Injury") or with no treatment ("Control"). Survival is shown as Kaplan-Meier curves for each stress treatment and protein restriction diets (A). For ease of interpretation, a subset of protein restriction diets is shown in (B) to illustrate the effects of protein restriction with low (yellow and green lines), intermediate (light blue lines) and high protein content (dark blue and black lines). Survival was maximized on intermediate protein across all stress treatments, as survival was poor on low (yellow line) and high protein diets (black line). Plus signs (+) indicate censored data points.

Table S2: Model summary of effects of protein restriction and stress treatments on mortality risk per day from an event history binomial model. In the binomial model, per each fly for each day, 0 coded for flies alive and 1 for dead. Protein and protein^2 are mean centered to standard deviation of 1. The model included random effects of Individual ID (posterior mean = 0.03 (95% credible interval (CI) = 7.56×10^{-10} to 0.11), effective sample size = 1013) and Experimental day (posterior mean = 2.38 (95% CI = 1.61 to 3.25), effective sample size = 1000). Significant results below significance level $\alpha = 0.05$ are bolded.

	Posterior mean	l-95% CI	u-95% CI	Effective sample size	pMCMC
**Intercept**	**-5.46**	**-5.89**	**-5.08**	**1000**	**<0.001**
Injury treatment	0.14	-0.32	0.57	1000	0.54
**Infection treatment**	**0.66**	**0.28**	**1.10**	**1000**	**0.002**
Protein	0.02	-0.13	1.17	1000	0.82
**Protein^2**	**0.48**	**0.26**	**0.71**	**1111**	**<0.001**
Injury:Protein	-0.08	-0.30	0.15	1000	0.45
**Infection:Protein**	**-0.31**	**-0.57**	**-0.10**	**1000**	**0.004**
Injury:Protein^2	-0.16	-0.51	0.18	1000	0.36
Infection:Protein^2	-0.01	-0.33	0.30	1000	0.99

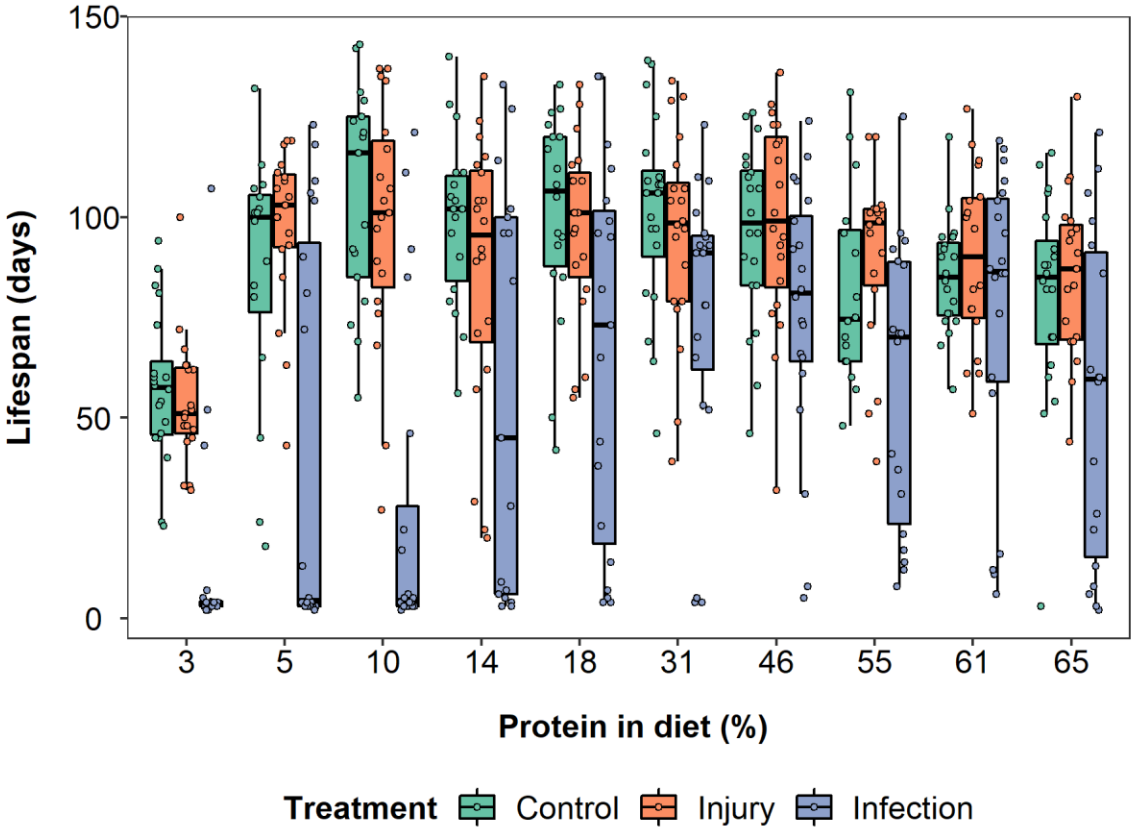

Figure S3: Effects of protein restriction on the lifespan of flies infected with a bacterial pathogen (blue bars and data points), injured by a pinprick (orange bars and data points) or with no treatment (green bars and data points). Data are observed lifespans (filled circles), where lines in the box plots indicate median lifespan (50% quantile), boxes are the interquartile range (25% to 75% quantiles) and whiskers are minimum or maximum quartiles (25% - 1.5 x interquartile range, 75% + 1.5 x interquartile range).

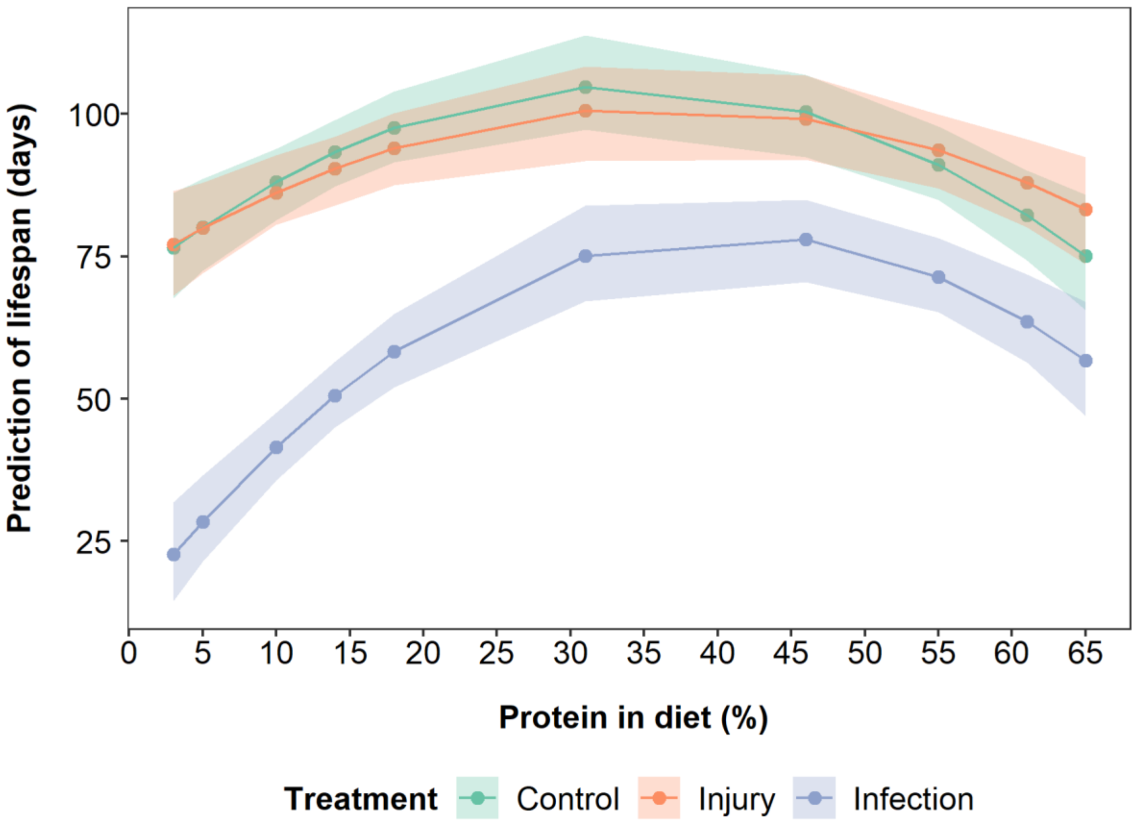

Figure S4: Model predictions of the effects of protein restriction on lifespan of flies infected with a bacterial pathogen (blue data points and lines), injured by a pinprick (orange data points and lines) or with no treatment (green data points and lines). Shaded areas are 95% 95% credible intervals. Protein and protein^2 are mean centered to standard deviation of 1.

Table S3: Model summary of effects of protein restriction and stress treatments on lifespan. Protein and protein^2 are mean centered to standard deviation of 1. The model included random effects of Individual ID (posterior mean = 0.028 (7.56 x 10^{-10}-0.11), effective sample size = 1013) and Experimental day (posterior mean = 2.38 (1.61-3.25), effective sample size = 1000). Significant results below significance level $\alpha = 0.05$ are bolded.

	Posterior mean	l-95% CI	u-95% CI	Effective sample size	pMCMC
**Intercept**	**104.76**	**97.22**	**113.77**	**1000**	**<0.001**
Injury treatment	-4.17	-15.32	7.55	1107	0.48
**Infection treatment**	**-29.83**	**-41.32**	**-17.72**	**1000**	**<0.001**
Protein	3.83	-0.16	-9.21	1000	0.09
**Protein^2**	**-15.79**	**-22.55**	**-8.90**	**1108**	**<0.001**
Injury:Protein	1.57	-5.97	7.50	1000	0.65
**Infection:Protein**	**14.31**	**7.66**	**20.99**	**1000**	**<0.001**
Injury:Protein^2	4.47	-5.33	14.45	1158	0.39
Infection:Protein^2	-4.44	-14.45	6.06	1000	0.40

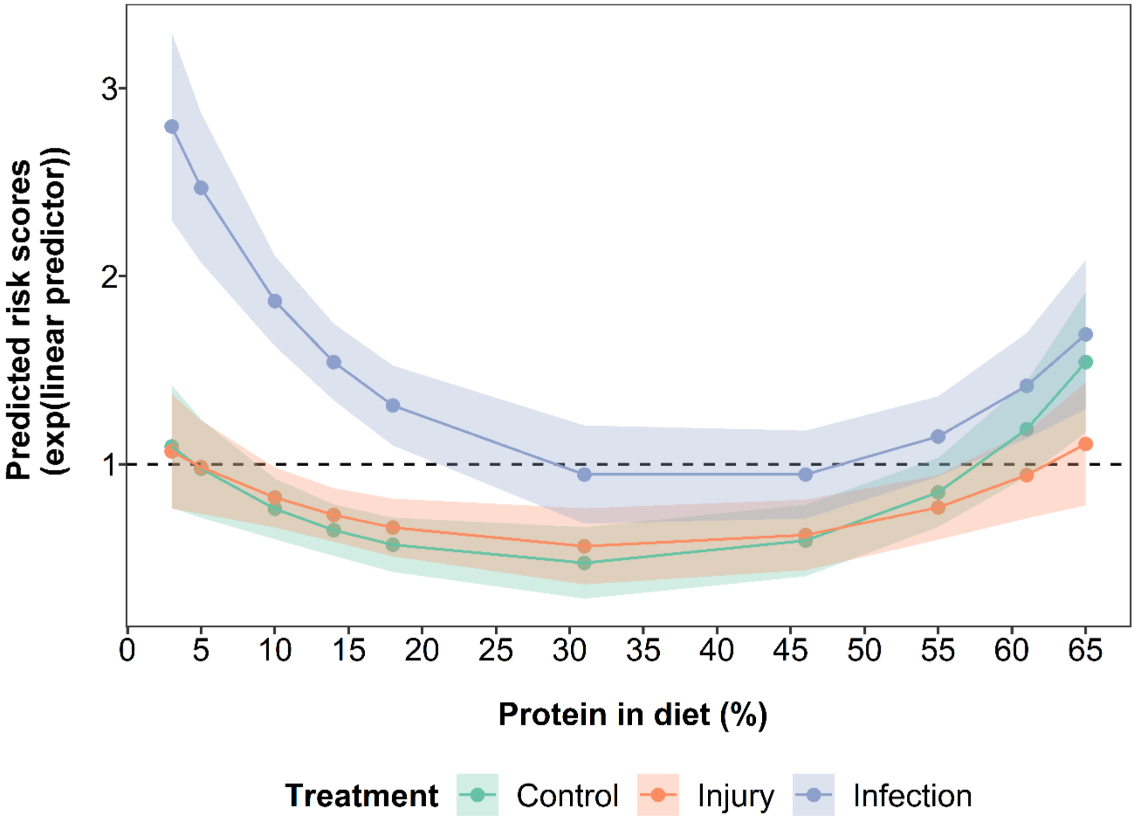

Figure S5: Model predictions for the effects of protein restriction on survival of flies infected with a bacterial pathogen (blue data points and lines), injured by a pinprick (orange data points and lines) or with no treatment (green data points and lines). $y = 1$ line shows no change in risk ratio, i.e. treatment would have no effect compared to baseline hazard. Shaded areas are 95% confidence intervals. Protein and protein^2 are mean centered to standard deviation of 1.

Table S4: Cox proportional hazard regression model summary of effects of protein restriction and stress treatments on survival (n = 600, number of deaths = 573, concordance = 0.662, $R^2 = 0.142$, Wald test = 97.98). Protein and protein^2 are mean centered to standard deviation of 1. Significant results below significance level $\alpha = 0.05$ are bolded.

	coef	exp(coef)	se(coef)	z	Pr (>|z|)
Injury treatment	0.17	1.19	0.20	0.84	0.40
**Infection treatment**	**0.69**	**1.99**	**0.21**	**3.36**	**<0.001**
Protein	-0.03	0.97	0.09	-0.29	0.77
**Protein^2**	**0.54**	**1.72**	**0.12**	**4.28**	**<0.001**
Injury:Protein	-0.06	0.94	0.12	-0.52	0.60
**Infection:Protein**	**-0.29**	**0.75**	**0.12**	**-2.43**	**0.01**
Injury:Protein^2	-0.18	0.83	0.18	-1.03	0.30
Infection:Protein^2	-0.07	0.93	0.18	-0.41	0.68

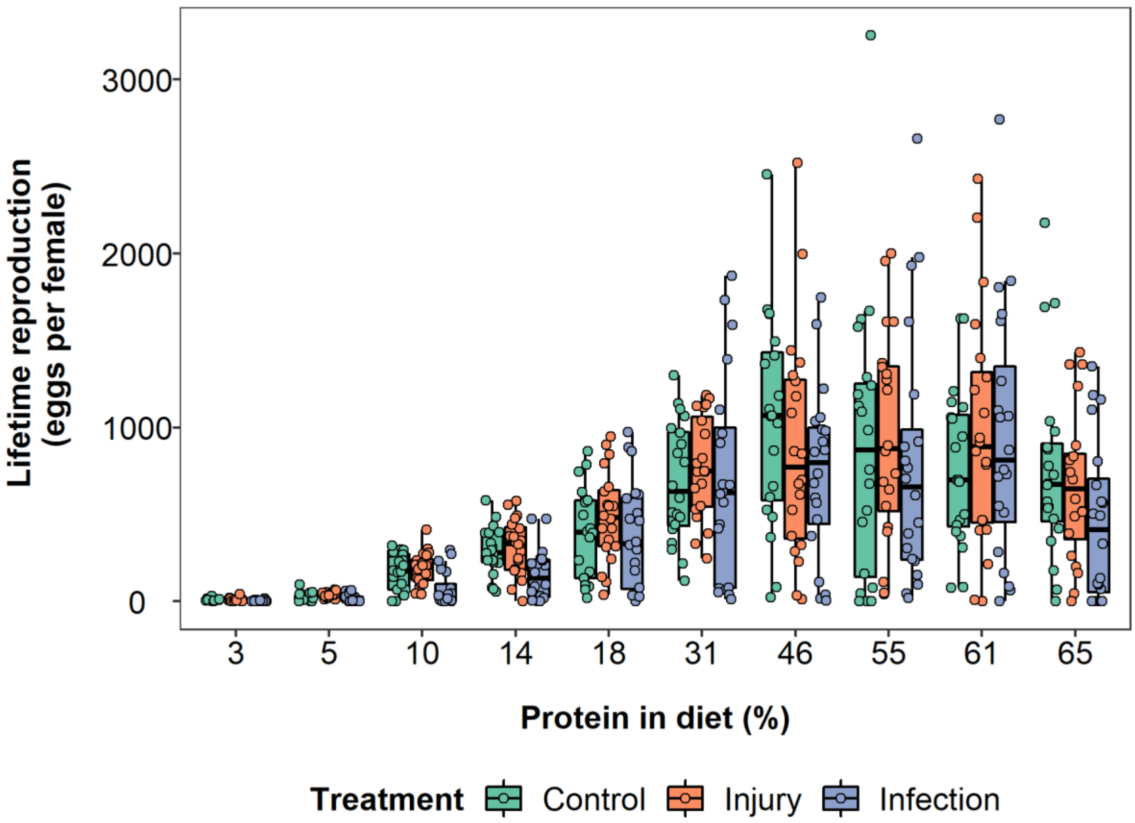

Figure S6: Effect of protein restriction on the lifetime egg production of flies infected with a bacterial pathogen (blue lines and data points), injured by a pinprick (orange lines and data points) or with no treatment (green lines and data points). The lines in the box plots indicate median lifespan (50% quantile), the boxes are the interquartile range (25% to 75% quantiles) and the whiskers are minimum or maximum quartiles (25% - 1.5 x interquartile range, 75% + 1.5 x interquartile range).

Table S5: Model summary of effects of protein restriction and stress treatments on lifetime eggs produced. Protein and protein^2 are mean centered to standard deviation of 1. Significant results below significance level $\alpha = 0.05$ are bolded.

	Posterior mean	l-95% CI	u-95% CI	Effective sample size	pMCMC
**Intercept**	**6.55**	**6.17**	**6.92**	**1205**	**<0.001**
Injury treatment	0.19	-0.34	0.72	1000	0.49
Infection treatment	-0.33	-0.90	0.16	1330	0.264
**Protein**	**1.45**	**1.23**	**1.64**	**1000**	**<0.001**
**Protein^2**	**-1.36**	**-1.68**	**-1.02**	**1000**	**<0.001**
Injury:Protein	-0.09	-0.39	-1.02	1000	0.60
**Infection:Protein**	**0.47**	**0.16**	**0.77**	**1000**	**0.01**
Injury:Protein^2	-0.02	-0.45	0.46	1000	0.93
**Infection:Protein^2**	**-0.47**	**-0.93**	**-0.04**	**1000**	**0.04**

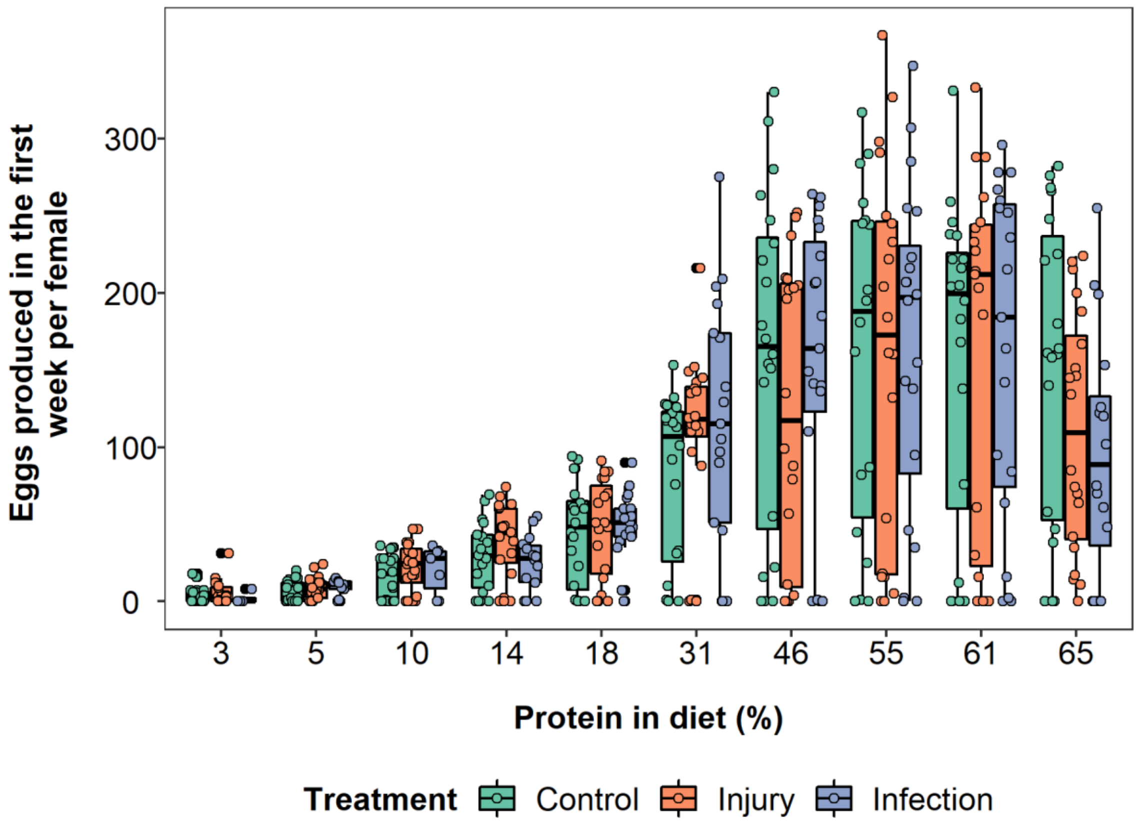

Figure S7: Effect of protein restriction on the early-life egg production of flies infected with a bacterial pathogen (blue lines and data points), injured by a pinprick (orange lines and data points) or with no treatment (green lines and data points). Early-egg production consists of the first seven days of egg production without the first day (see methods). The lines in the box plots indicate median number of eggs produced (50% quantile), boxes are the interquartile range (25% to 75% quantiles) and whiskers are minimum or maximum quartiles (25% - 1.5 x interquartile range, 75% + 1.5 x interquartile range).

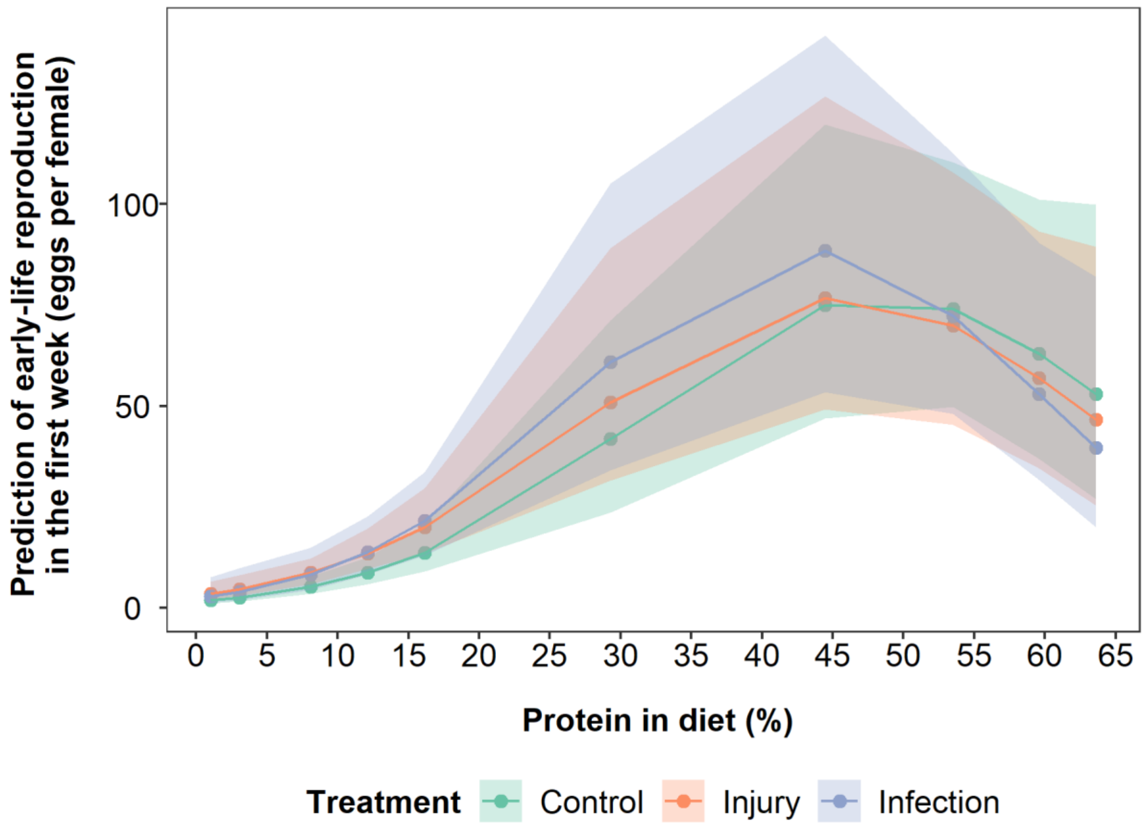

Figure S8: Model predictions of the effect of protein restriction on the early-life egg production of flies infected with a bacterial pathogen (blue data points and lines), injured by a pinprick (orange data points and lines) or with no treatment (green data points and lines). Early-life egg production consists of the first seven days of egg production without the first day (see methods). Shaded areas are 95% 95% credible intervals. Protein and protein^2 are mean centered to standard deviation of 1.

Table S6: Model summary of effect of protein restriction and stress treatment on early-life egg production (first week discounting the first day, see methods). Protein and protein^2 are mean centered to standard deviation of 1. Significant results below significance level $\alpha = 0.05$ are bolded.

	Posterior mean	l-95% CI	u-95% CI	Effective sample size	pMCMC
**Intercept**	**3.82**	**3.30**	**4.41**	**1156**	**<0.001**
Injury treatment	0.18	-0.56	0.92	1000	0.69
Infection treatment	0.36	-0.37	1.16	1330	0.37
**Protein**	**1.34**	**1.06**	**1.64**	**1000**	**<0.001**
**Protein^2**	**-0.86**	**-1.34**	**-0.41**	**1149**	**<0.001**
Injury:Protein	-0.29	-0.69	0.10	1000	0.17
Infection:Protein	-0.24	-0.74	0.20	1000	0.32
Injury:Protein^2	0.05	-0.60	0.66	1000	0.86
Infection:Protein^2	-0.14	-0.85	0.57	1198	0.74

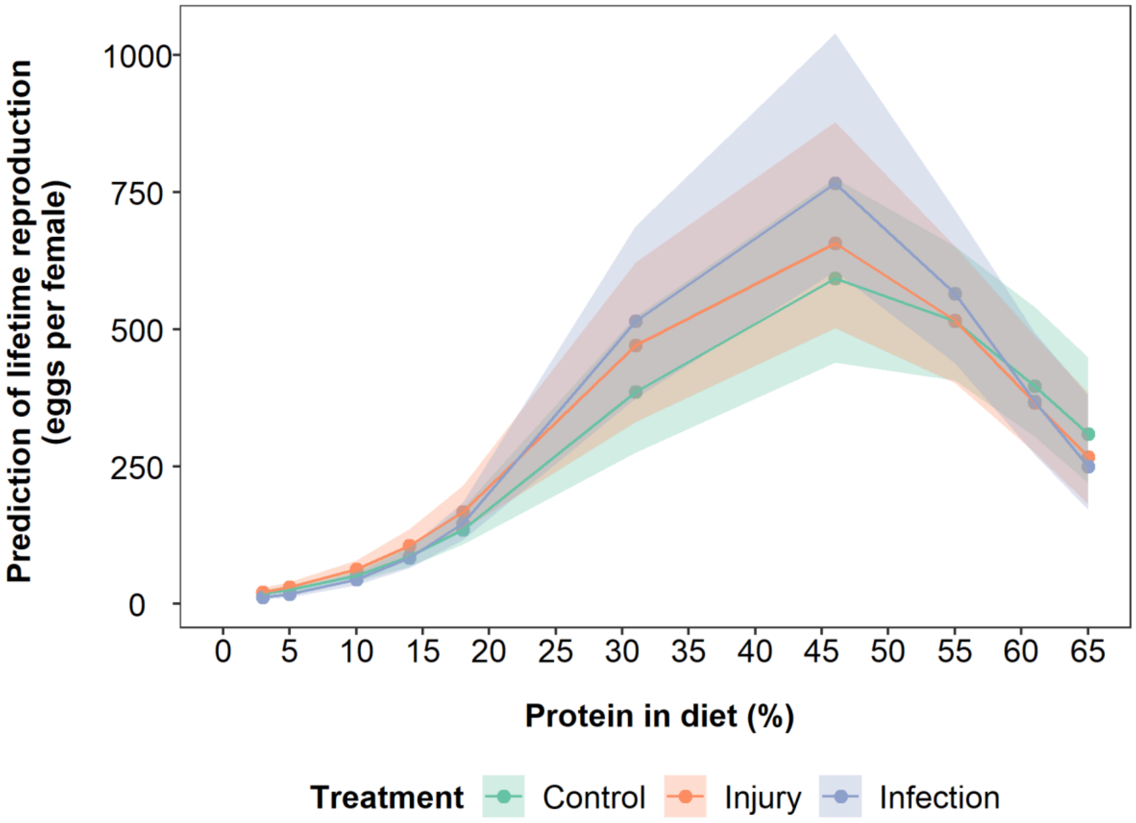

Figure S9: Model predictions of the effects of protein restriction on the lifetime number of eggs produced by flies infected with a bacterial pathogen (blue data points and lines), injured by a pinprick (orange data points and lines) or with no treatment (green data points and lines), when accounting for lifespan (mean centred). Shaded areas are 95% credible intervals. Protein and protein^2 are mean centered to standard deviation of 1.

Table S7: Model summary of effects of protein restriction and stress treatments on lifetime eggs produced. Mean centered lifespan is added as a fixed effect to remove the effect of lifespan on reproduction. Protein and protein^2 are mean centered to standard deviation of 1. Significant results below significance level $\alpha = 0.05$ are bolded.

	Posterior mean	l-95% CI	u-95% CI	Effective sample size	pMCMC
**Intercept**	**5.94**	**5.64**	**6.28**	**1000**	**<0.001**
Injury treatment	0.20	-0.19	0.67	1000	0.36
Infection treatment	0.29	-0.18	0.72	1000	0.21
**Protein**	**1.31**	**1.15**	**1.50**	**1119**	**<0.001**
**Protein^2**	**-0.97**	**-1.24**	**-0.70**	**1000**	**<0.001**
**Lifespan**	**0.93**	**0.83**	**1.04**	**1000**	**<0.001**
Injury:Protein	-0.81	-0.33	0.17	1000	0.52
Infection:Protein	0.18	-0.07	0.45	1000	0.17
Injury:Protein^2	-0.10	-0.45	0.28	1000	0.60
Infection:Protein^2	-0.34	-0.71	0.05	1000	0.08

AGEING:

<u>DAILY EGG PRODUCTION:</u>

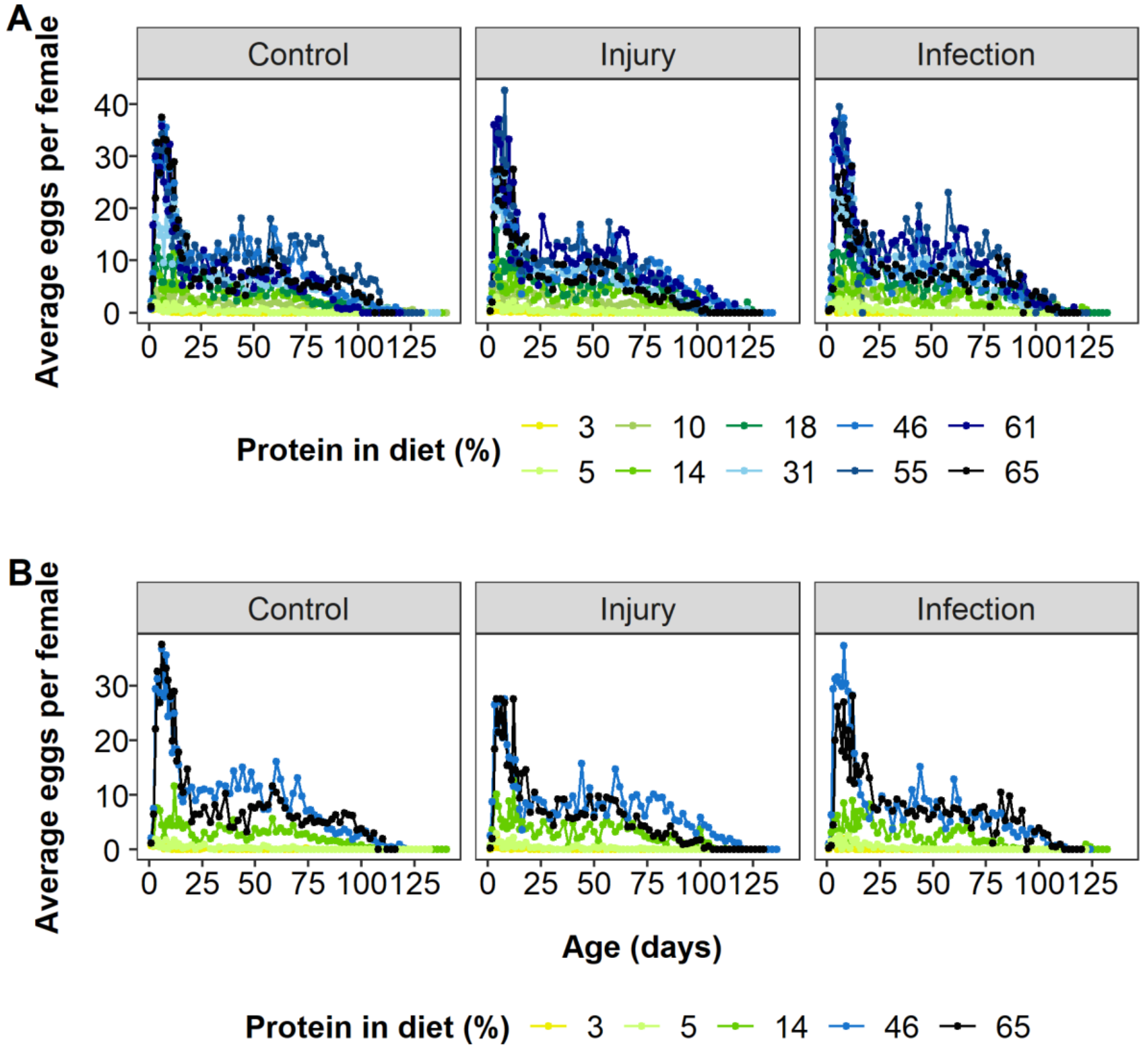

Figure S10: The pattern of ageing in egg production for each protein restriction diet for flies infected with a bacterial pathogen ("Infection"), injured by a pinprick ("Injury") or with no treatment ("Control"). The average eggs laid per day across all flies per diet and stress treatment is plotted and the associated errors have been removed for clarity. (A) All diets for each stress treatment; (B) A subset of protein restriction diets to illustrate the effects of protein restriction with low (yellow line) intermediate (blue line) and high protein content (black line).

Table S8: Model summary of effects of protein restriction, age and stress treatment for daily egg production on flies. Protein, protein^2, age, age^2 and lifespan are mean centered to standard deviation of 1. The model included random effects of Individual ID (posterior mean = 1.76 (95% CI = 1.53 to 2.01), effective sample size = 1334). Lifespan (mean centered) is included to account for selective disappearance. Significant results below significance level α = 0.05 are bolded.

	Posterior mean	l-95% CI	u-95% CI	Effective sample size	pMCMC
**Intercept**	**1.61**	**1.23**	**1.98**	**1334**	**<0.001**
Injury treatment	0.19	-0.35	0.68	1193	0.48
Infection treatment	-0.11	-0.66	0.47	1334	0.72
**Protein**	**1.31**	**1.12**	**1.52**	**1334**	**<0.001**
**Protein^2**	**-1.51**	**-1.81**	**-1.19**	**1334**	**<0.001**
**Age**	**-0.32**	**-0.40**	**-0.23**	**1222**	**<0.001**
**Age^2**	**-0.52**	**-0.59**	**-0.44**	**1334**	**<0.001**
**Lifespan**	**0.21**	**0.11**	**0.31**	**1334**	**<0.001**
Injury:Protein	0.08	-0.22	0.35	1334	0.58
Infection:Protein	-0.21	-0.54	0.11	1334	0.22
Injury:Protein^2	-0.05	-0.51	0.36	1477	0.82
**Infection:Protein^2**	**0.51**	**0.04**	**0.97**	**1334**	**0.03**
**Injury:Age**	**0.13**	**0.01**	**0.24**	**1334**	**0.03**
**Infection:Age**	**-0.29**	**-0.42**	**-0.14**	**1334**	**<0.001**

Injury:Age^2	0.08	-0.03	0.19	1063	0.15
**Infection:Age^2**	**0.27**	**0.13**	**0.41**	**1334**	**0.002**
Protein:Age	0.02	-0.04	0.08	1334	0.56
Protein:Age^2	-0.04	-0.09	0.02	1660	0.22
**Protein^2:Age**	**-0.24**	**-0.32**	**-0.16**	**1334**	**<0.001**
**Protein^2:Age^2**	**0.30**	**0.22**	**0.38**	**1116**	**<0.001**
**Injury:Protein:Age**	**0.14**	**0.05**	**0.22**	**1334**	**0.002**
**Infection:Protein:Age**	**0.11**	**0.001**	**0.20**	**1334**	**0.04**
**Injury:Protein:Age^2**	**-0.14**	**-0.22**	**-0.06**	**1334**	**<0.001**
Infection:Protein:Age^2	0.02	-0.09	0.13	1334	0.64
**Injury:Protein^2:Age**	**-0.12**	**-0.23**	**-0.005**	**1360**	**0.04**
**Infection:Protein^2:Age**	**0.22**	**0.09**	**0.35**	**1334**	**0.005**
**Injury:Protein^2:Age^2**	**-0.13**	**-0.25**	**-0.02**	**1175**	**0.03**
**Infection:Protein^2:Age^2**	**-0.37**	**-0.51**	**-0.23**	**1334**	**<0.001**

SMURF ASSAY:

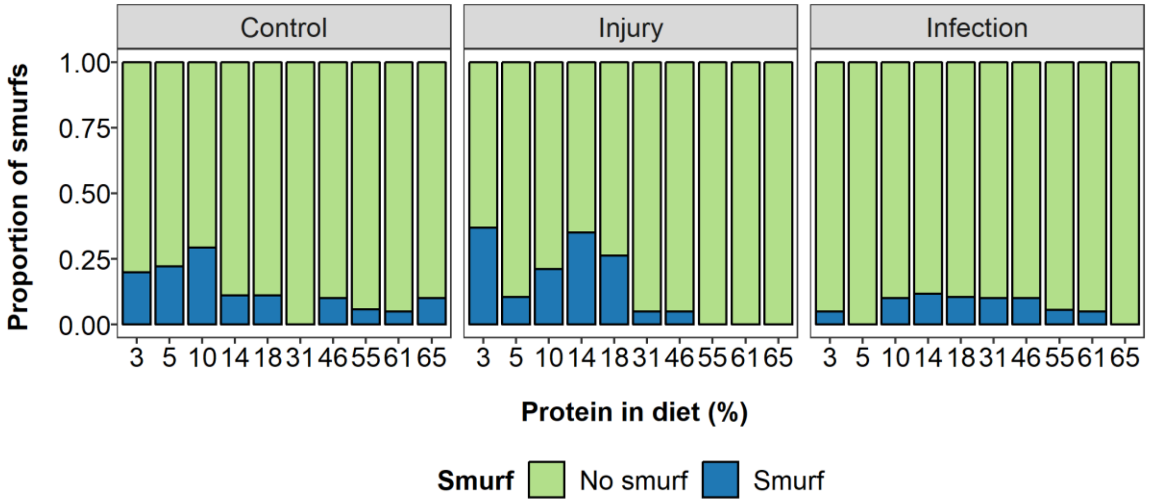

Figure S11: Effects of protein restriction on proportion of smurfs (blue bars) or no smurfs (green bars) across life of flies infected with a bacterial pathogen ("Infection", N = 23), injured by a pinprick ("Injury", N = 25) or with no treatment ("Control", N = 15).

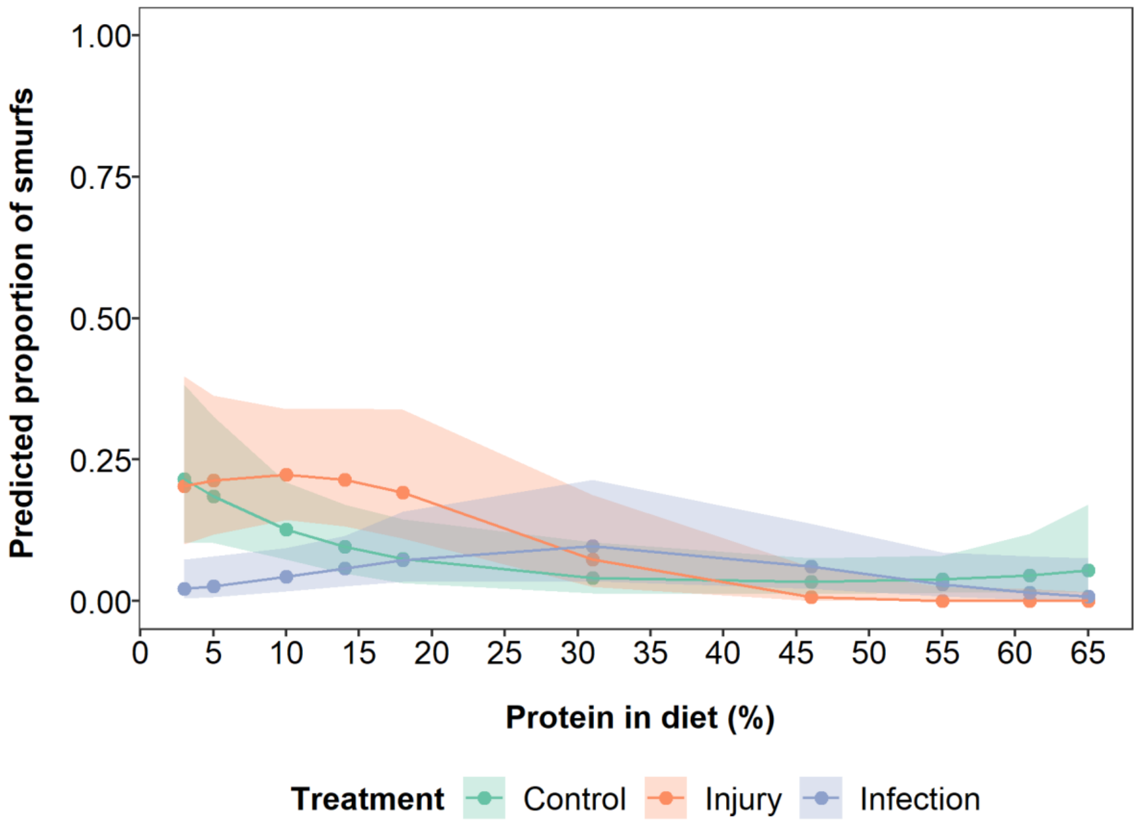

Figure S12: Model predictions of the effect of protein restriction on the proportion of flies developing into a smurf of flies infected with a bacterial pathogen (blue data points and lines), injured by pinprick (orange data points and lines) or with no treatment (green data points and lines). Protein and protein^2 are mean centered to standard deviation of 1. Shaded areas are 95% credible intervals.

Table S9: Model summary of effects of protein restriction and stress treatment on proportion of flies developing into a smurf. Protein and protein^2 are mean centered to standard deviation of 1. Significant results below significance level $\alpha = 0.05$ are bolded.

	Posterior mean	l-95% CI	u-95% CI	Effective sample size	pMCMC
**Intercept**	**-3.13**	**-4.21**	**-2.07**	**1000**	**<0.001**
Injury treatment	0.63	-0.81	2.27	1000	0.37
Infection treatment	0.91	-0.59	2.30	1000	0.23
**Protein**	**-0.75**	**-1.24**	**-0.21**	**1000**	**0.004**
Protein^2	0.63	-0.26	1.41	1000	0.15
**Injury:Protein**	**-1.96**	**-4.07**	**-0.11**	**1000**	**0.01**
Infection:Protein	0.73	-0.20	1.71	1000	0.10
**Injury:Protein^2**	**-2.09**	**-4.22**	**-0.47**	**1000**	**0.14**
**Infection:Protein^2**	**-1.73**	**-3.13**	**-0.37**	**1000**	**0.01**

NEGATIVE GEOTAXIS (NG):

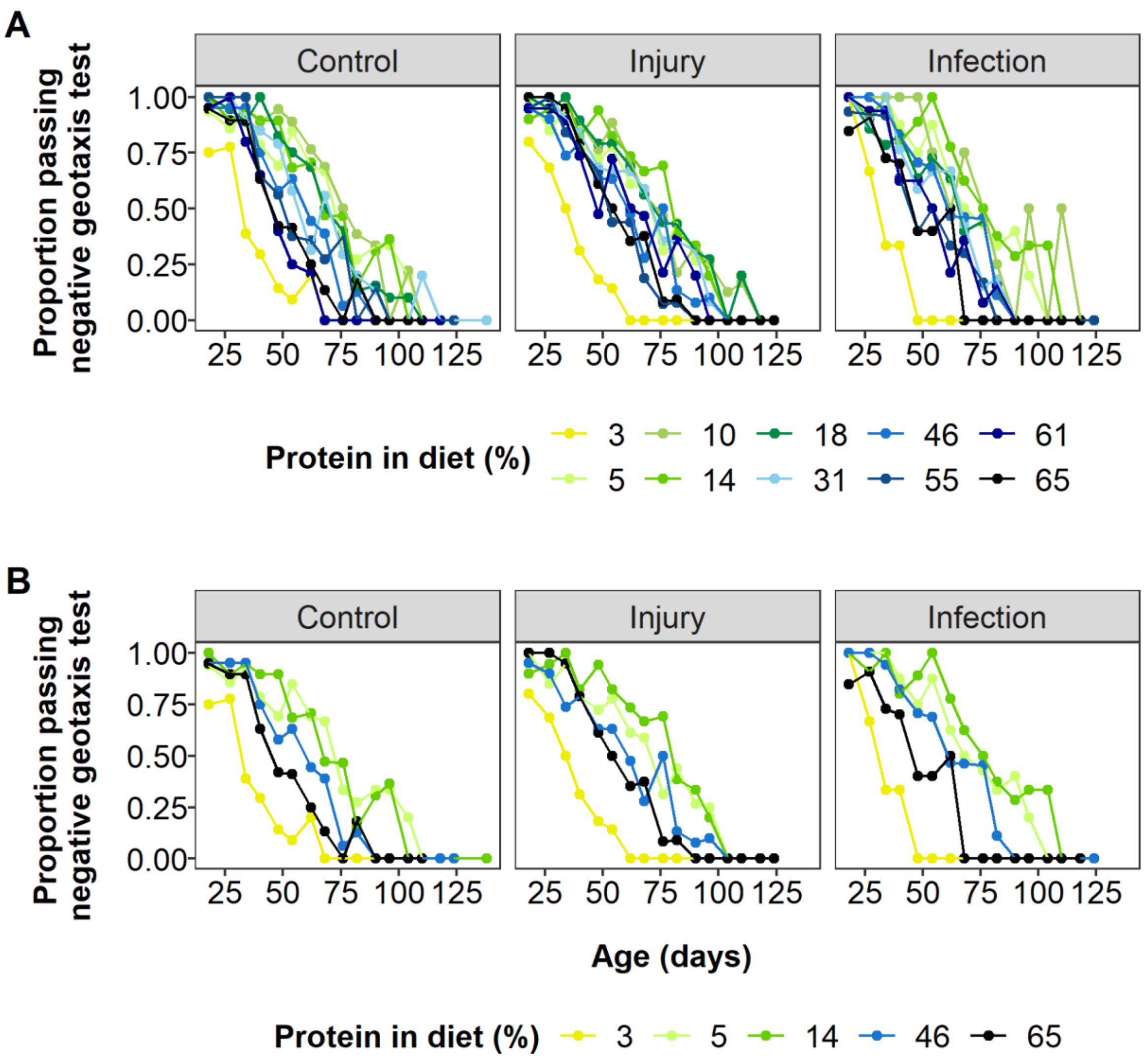

Figure S13: Effects of protein restriction on the proportion of flies passing the negative geotaxis test under 60 seconds per week of flies infected with a bacterial pathogen ("Infection"), injured by a pinprick ("Injury"), or with no treatment ("Control") (A). For ease of interpretation, a subset of diets is shown in (B) to illustrate the effects of protein restriction with low (yellow line) intermediate (pale blue line) and high protein content (black line).

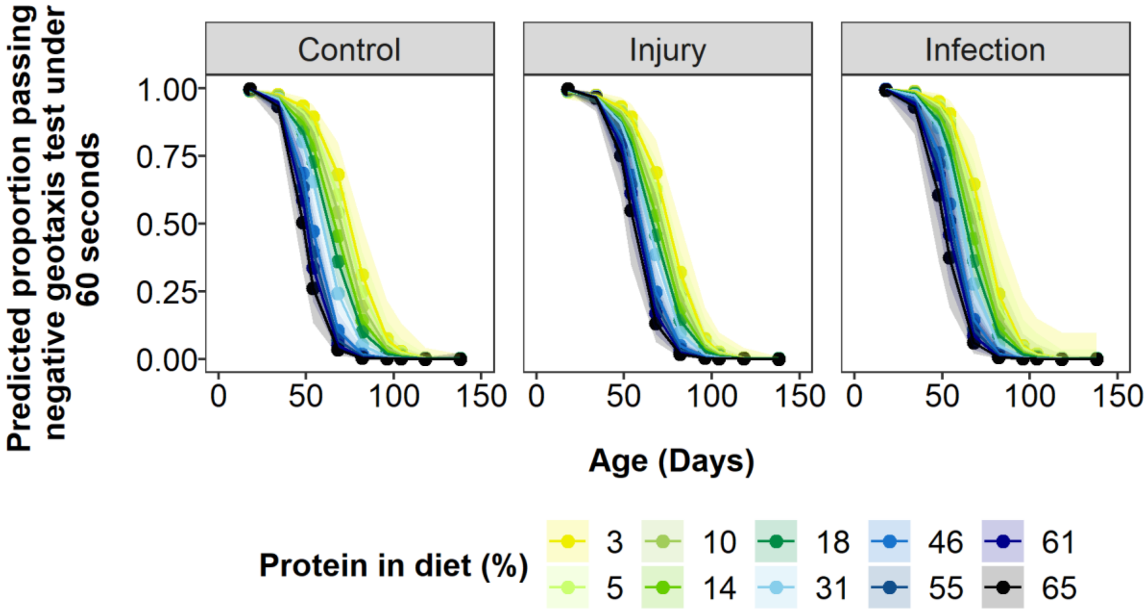

Figure S14: Model predictions of the effect of protein restriction and age on proportion passing negative geotaxis test under 60 seconds per week with flies infected with a bacterial pathogen ("Infection"), injured by pinprick ("Injury") or with no treatment ("Control"). Shaded areas are 95% credible intervals. Protein, protein^2 and lifespan are mean centered to standard deviation of 1.

Table S10: Model summary of effects of protein restriction, age and stress treatment for passing negative geotaxis test under 60 seconds. Protein, protein^2, age, age^2 and lifespan are mean centered to standard deviation of 1. Lifespan (mean centered) is included to account for selective disappearance. The model included the random effect of Individual ID (posterior mean = 3.03 (95% CI = 2.35 to 3.73), effective sample size = 1000). Significant results below significance level α = 0.05 are bolded.

	Posterior mean	l-95% CI	u-95% CI	Effective sample size	pMCMC
**Intercept**	**1.01**	**0.39**	**1.63**	**892.4**	**0.002**
Injury treatment	0.52	-0.38	1.37	1000	0.23
Infection treatment	0.35	-0.59	1.32	1000	0.49
**Protein**	**-0.65**	**-1.01**	**-0.32**	**1000**	**<0.001**
**Protein^2**	**-0.70**	**-1.21**	**-0.21**	**1060**	**0.01**
**Age**	**-3.57**	**-4.04**	**-3.07**	**1000**	**<0.001**
Age^2	-0.13	-0.61	0.36	902.6	0.58
**Lifespan**	**0.84**	**0.64**	**1.02**	**1000**	**<0.001**
Injury:Protein	0.38	-0.07	0.86	1197.5	0.12
Infection:Protein	0.20	-0.40	0.81	1000	0.53
Injury:Protein^2	0.10	-0.60	0.78	1102.8	0.78
Infection:Protein^2	-0.04	-0.76	0.89	1101.4	0.93
Injury:Age	0.48	-0.15	1.17	1098.6	0.17

Infection:Age	-0.17	-1.03	0.51	1039.8	0.69
Injury:Age^2	-0.30	-0.86	0.43	1000	0.37
Infection:Age^2	-0.26	-1.12	0.47	1000	0.51
**Protein:Age**	**-0.78**	**-1.06**	**-0.49**	**1108.6**	**<0.001**
Protein:Age^2	0.16	-0.13	0.42	1000	0.27
$Protein^2$:Age	0.06	-0.33	0.48	1124.5	0.79
$Protein^2$:Age^2	0.15	-0.28	0.50	1000	0.45
Injury:Protein:Age	0.28	-0.11	0.63	1000	0.16
Infection:Protein:Age	0.37	-0.10	0.91	1132.2	0.17
Injury:Protein:Age^2	-0.17	-0.54	0.19	1045.3	0.40
Infection:Protein:Age^2	-0.35	-0.83	0.10	1227	0.14
Injury:$Protein^2$:Age	-0.17	-0.72	0.39	1000	0.54
Infection:$Protein^2$:Age	-0.03	-0.63	0.75	1000	0.95
Injury:$Protein^2$:Age^2	0.09	-0.45	0.60	1000	0.73
Infection:$Protein^2$:Age^2	0.13	-0.56	0.80	1000	0.69
